# Integration of HIV and reproductive health services in public sector facilities: analysis of client flow data over time in Kenya

**DOI:** 10.1136/bmjgh-2018-000867

**Published:** 2018-09-14

**Authors:** Isolde J Birdthistle, Justin Fenty, Martine Collumbien, Charlotte Warren, James Kimani, Charity Ndwiga, Susannah Mayhew, Integra Initiative, Susannah Mayhew

**Affiliations:** 1 Department of Population Health, London School of Hygiene & Tropical Medicine, London, UK; 2 Department of Global Health and Development, London School of Hygiene & Tropical Medicine, London, UK; 3 Population Council, Washington, District of Columbia, USA; 4 Population Council, Nairobi, Kenya

**Keywords:** HIV, child health, health services research, prevention strategies, intervention study

## Abstract

**Introduction:**

Integration of HIV/AIDS with reproductive health (RH) services can increase the uptake and efficiency of services, but gaps in knowledge remain about the practice of integration, particularly how provision can be expanded and performance enhanced. We assessed the extent and nature of service integration in public sector facilities in four districts in Kenya.

**Methods:**

Between 2009 and 2012, client flow assessments were conducted at six time points in 24 government facilities, purposively selected as intervention or comparison sites. A total of 25 539 visits were tracked: 15 270 in districts where 6 of 12 facilities received an intervention to strengthen HIV service integration with family planning (FP); and 10 266 visits in districts where half the facilities received an HIV-postnatal care intervention in 2009–2010. We tracked the proportion of all visits in which: (1) an HIV service (testing, counselling or treatment) was received together with an RH service (FP counselling or provision, antenatal care, or postnatal care); (2) the client received HIV counselling.

**Results:**

Levels of integrated HIV-RH services and HIV counselling were generally low across facilities and time points. An initial boost in integration was observed in most intervention sites, driven by integration of HIV services with FP counselling and provision, and declined after the first follow-up. Integration at most sites was driven by temporary rises in HIV counselling. The most consistent combination of HIV services was with antenatal care; the least common was with postnatal care.

**Conclusions:**

These client flow data demonstrated a short-term boost in integration, after an initial intervention with FP services providing an opportunity to expand integration. Integration was not sustained over time highlighting the need for ongoing support. There are multiple opportunities for integrating service delivery, particularly within antenatal, FP and HIV counselling services, but a need for sustained systems and health worker support over time.

**Trial registration number:**

NCT01694862

**Key questions**

**What is already known?**

- Clients and facilities can benefit from integrating HIV/AIDS services with reproductive health, maternal health and child health services.

**What are the new findings?**

- With detailed client-level time-series data (25 539 client visits) on receipt of integrated services, this study adds important insights into how integrated service delivery is being rolled out in ‘real-world’ settings and how it can be supported and expanded in the public sector facilities in Kenya.
- A short-term boost in integration of HIV services with family planning counselling and provision was not sustained in contrast to the more consistent integration of HIV services and antenatal care.
- HIV counselling appears to act as the glue or linking service with core reproductive health services offering a key entry point for integrated delivery of family planning and HIV services.

**What do the new findings imply?**

- To sustain integration of HIV services with family planning counselling and provision, prioritisation and funding commitment similar to that enjoyed by prevention of mother-to-child HIV transmission initiatives is needed.

## Introduction

A strong case has been made for the potential benefits of integrating HIV/AIDS services and standard care services like reproductive health (RH), maternal health and child health services. Clients and facilities can benefit, through increased uptake, quality and efficiency of services.^1–5^ A scoping study in 2014 found that integration is supported in major international health^6^ policies, national strategies and donor guidance,^7^ yet knowledge gaps remained about actual levels and performance of integration in public sector facilities, or how provision can be improved and scaled up.^3^ The authors called for rigorous health systems research on the integration of HIV services with sexual and RH services in sub-Saharan Africa, to inform the delivery of integrated services.

Since then, research—including two journal supplements on integration of RH and HIV services—has widened the knowledge base (most recently, the *Health Policy and Planning* supplement, 2017). Integration at policy level remains surprisingly weak^8^ and the need for systems-wide approaches to scale-up of integrated delivery of care is clear.^9–12^ Studies assessing systems considerations for HIV-service integration show the need for collaboration and coordination between teams, staff and patients,^12–14^ and the need to invest in the health workforce, particularly to support agency of decision-making, team working and load sharing.^11 12 14 15^

Studies on service delivery have shown that integration of family planning (FP) into HIV services in Kenya can improve uptake of contraception (other than condoms)^6^ and can expand access to cervical cancer screening and prevention of mother-to-child HIV transmission (PMTCT).^16^ Integrating HIV testing into FP services can improve the WHO’s recommended testing rates for HIV among FP clients where women are exposed to well-integrated services.^17^ Integration of HIV and postnatal care (PNC) services was found to increase provider-initiated counselling and testing and uptake of long-acting FP methods among postpartum women.^18^ Integrated health services could also play a role in combatting intimate partner violence.^19 20^

What is still lacking in evidence is the extent to which public sector services are able to deliver integrated services in practice and which combinations of integrated services are provided on a regular basis. The Integra Initiative was designed to evaluate different models of integration in Kenya and Swaziland and collected data on thousands of client visits over a 2-year period to determine integration patterns.^21^ An analysis of the client flow data in eight government facilities in Swaziland found that provision of HIV and sexually transmitted infection (STI) services with maternal, reproductive and child healthcare occurred at all facilities, yet only a small minority of women received integrated services.^22^ Four of the facilities showed increases in overall integration between 2010 and 2012, driven primarily by increases in HIV counselling. Specifically, HIV counselling was most often integrated with child health services, antenatal care (ANC) or FP, and least often with PNC. Sharp declines in integration over time suggested that integration is difficult to sustain, and hindered by factors such as frequent staff rotation and vertical HIV/AIDS campaigns, for example, for testing or treatment.

This study analyses client flow data collected in 24 public sector facilities in Kenya between 2009 and 2012. We track whether clients received integrated services, and if so, in what combinations. We also describe how the receipt of integrated services differs over time and between facilities.

## Methods

### Data collection

#### Integration of HIV and PNC services

Two districts with similar characteristics (in the area known as Eastern Province prior to devolution in 2010) were designated by the Ministry of Health. In one district—randomly selected to receive the Intervention—six public sector facilities were purposively designated, to include a range of settings and facility types. They are referred to collectively as ‘District E1’ facilities and individually as Facilities A–F. In the other district, six comparison facilities were selected based on their distance from intervention sites, to avoid contamination, and no current provision of integrated HIV-PNC services (Facilities G–L, or ‘District E2’ collectively). To maximise comparability, non-intervention sites were selected based on similarities with the intervention sites in terms of client load, number of providers, health infrastructure and the socioeconomic profile of clients.

In the intervention facilities, between August 2009 and December 2010, Integra delivered a programme designed to strengthen and maintain the provision of integrated HIV/STI and PNC services. The intervention components included: (A) a training package, comprising 16 lessons, to facilitate mentoring of front-line health providers by more experienced providers in each facility; (B) job aids to promote integration, including the Balanced Counselling Strategy Plus toolkit containing an algorithm, counselling cards and brochures to support counselling, including HIV service provision, within postnatal consultations^23 24^; and (C) ongoing, on-site supportive supervision, provided quarterly and jointly by HIV and RH coordinators of the district Ministry of Health, to discuss role clarification, organisational change, referral/linkages and management of service statistics. Ongoing support involved troubleshooting and identifying gaps in service provision, supplies and equipment. Further details about implementation of the intervention are described in the published protocol for the Integra^21^ Initiative and the training guides and toolkits are publicly available.^23^

#### Integration of HIV and FP services

In the predevolution Central Province, six public facilities were designated as Intervention facilities (referred to collectively as ‘District C1’ facilities and individually as Facilities a–f) to receive the Integra programme, in this case focused on the integration of HIV/STI and FP services, within the same time frame as the former Eastern Province (August 2009 to December 2010). The six facilities—two hospitals and four health centres—were selected from an original 23 participating in a previous FP integration study, with selection based on: (1) good performance in the previous study; and (2) high volume of FP clients (>100/month).^25^ Six comparison facilities (g–l; collectively ‘District C2’) were chosen from outside the original FP study districts, with criteria designed to maximise similarities and minimise contamination with the Integra intervention facilities, using the same criteria as in the former Eastern Province (described above).

#### Client flow assessments: methodology and timing

Client flow assessments (CFA)—one data component of the Integra evaluation—were designed to capture robust service utilisation patterns among clients. CFAs were conducted in all study facilities at six time points over 3 years: June/July 2009; January 2010; June 2010; January/February 2011; August 2011; and January 2012.

Over a period of 5 days, Monday through Friday, all clients entering the maternal and child health unit of a study facility were given a client flow form by teams of trained local researchers or service providers. (See online supplementary table 5 for a copy of the tool.) Clients carried the form throughout their visit, and each service provider completed the form in their consultation room/cubicle, indicating session start and end times, the service(s) received by the client and any referrals to other providers. While intended to be simultaneous in all facilities over the same 5-day period, this proved difficult logistically. The timing of CFAs across facilities was close but not always simultaneous, and this analysis is restricted to the first Monday through Friday on which data were collected. Online supplementary tables 1 and 2 present the dates of data collection at each facility, and the dates included and omitted from analysis.

10.1136/bmjgh-2018-000867.supp1 Supplementary file 1

### Protocol changes

During implementation of the Integra trial, national governments in Kenya and Swaziland formally adopted and accelerated implementation of integration of HIV and reproductive/maternal health services in public health facilities. Additionally, numerous donors and non-governmental organisations were scaling up activities in some of the comparison sites. This removed operational distinction in service provision between facilities in intervention and comparison arms of the Integra study. Consequently, our assessment of the primary outcome measures shifted from a comparison of study arms to describing the extent of integration and changes over time for individual facilities and the impact of exposure to clinics that did integrate well.^12 17 26^ Although we retain the original district distinctions here, the purpose of this paper is not to evaluate the intervention or its effect but to describe and interpret the patterns of integrated service delivery in government facilities of the four districts. Other than an acknowledgement of the immediate postintervention effect, this paper focuses on understanding the prevalence of integrated service delivery over time and which service components were integrated.

### Outcome measures and data analysis

A ‘visit’ (the unit of analysis) comprised all providers seen and services received in the same day for each client, as captured on the client assessment form. Clients were either a single adult (male or female) or an adult plus a child. The following primary and secondary outcomes were calculated for each facility and time point:

1. Receipt of integrated HIV-RH services, defined as the proportion of all visits in which a client receives any HIV or STI service, specifically, HIV testing, counselling or treatment; or STI counselling or testing and any of the following RH services: FP counselling or provision; PNC for mother or baby; cervical cancer screening; gynaecology; or ANC.
2. Receipt of HIV counselling, measured by the proportion of all visits in which a client receives HIV counselling. HIV counselling was selected because it is expected to be conducted regardless of women’s need for HIV testing or treatment which are not constant.

We also sought to describe which RH services were most commonly combined with HIV/STI services, by calculating the percentage of visits in which an HIV/STI service was received with each type of RH service, for example, with FP or PNC specifically. We examined changes over time in the proportion of visits receiving integrated HIV/STI and RH services (primary outcome) and HIV counselling (secondary outcome) separately for each facility. We calculated 95% CIs to estimate the range of plausible values of the underlying ‘true’ change in service integration since baseline.

## Results

Over six time points between 2009 and 2012, a total of 25 539 visits tracked in 24 facilities were included in this analysis: 10 266 in the former Eastern Province (see online supplementary table 1) and 15 270 in former Central Province (online supplementary table 2). The characteristics of the study facilities and the visits tracked at baseline are presented in table 1 (and at all other rounds in online supplementary tables 3 and 4). In Districts E1 and E2, the mean age of clients was 26–27 years; this was also the case in Districts C1–2 with the exception of three facilities in which clients were older, 30–33 years on average. Across facilities, almost all clients received at least one service during their visit; the majority received two or more services in the same visit, usually from one provider. In almost all facilities, the most common service received was a child health service, such as immunisations and weighing. FP and ANC were among the top three services received in most facilities.

10.1136/bmjgh-2018-000867.supp2 Supplementary file 2

10.1136/bmjgh-2018-000867.supp3 Supplementary file 3

**Table 1:** Characteristics of the study facilities and client visits at baseline (June/July 2009)

HIV-PNC model	District E1 facilities (visits n=1029)						District E2 facilities (visits n=967)					
	A	B	C	D	E	F	G	H	I	J	K	L
Visits tracked	390	209	89	135	106	100	202	235	115	87	221	107
Client category												
Adult (12+ years) only, %	168 (43.1)	73 (34.9)	49 (55.1)	43 (31.9)	37 (34.9)	28 (28)	109 (54)	56 (23.8)	10 (8.7)	47 (54)	82 (37.1)	15 (14)
Adult+child (%)	222 (56.9)	136 (65.1)	40 (44.9)	92 (68.1)	69 (65.1)	72 (72)	93 (46)	179 (76.2)	105 (91.3)	40 (46)	139 (62.9)	92 (86)
Client gender												
Male	0	0	0	0	0	0	0	0	0	0	0	0
Female (%)	390 (100)	209 (100)	89 (100)	135 (100)	106 (100)	100 (100)	202 (100)	235 (100)	115 (100)	87 (100)	221 (100)	107 (100)
Client age												
Mean (SD)	26.7 (7.3)	26.4 (7.5)	29.4 (9)	27 (6.9)	25.7 (6.6)	26.2 (6.8)	27.3 (6.6)	26.7 (6.3)	27.5 (7.3)	27.5 (6.7)	27.3 (6.8)	27.6 (6.8)
Services received per visit												
None (%)	1 (0.3)	8 (3.8)	0	0	0	0	0	0	0	0	0	0
One (%)	173 (44.4)	116 (55.5)	46 (51.7)	37 (27.4)	30 (28.3)	56 (56)	105 (52)	26 (11.1)	61 (53)	29 (33.3)	157 (71)	24 (22.4)
Two or more (%)	216 (55.4)	85 (40.7)	43 (48.3)	98 (72.6)	76 (71.7)	44 (44)	97 (48)	209 (88.9)	54 (47)	58 (66.7)	64 (29)	83 (77.6)
Visits in which ≥1 service received												
Mean (SD) services received	1.7 (0.8)	1.4 (0.5)	1.7 (0.9)	1.8 (0.6)	1.8 (0.6)	1.8 (1.2)	1.9 (1.2)	2.9 (1.1)	1.6 (0.8)	2.3 (1.3)	1.3 (0.5)	2.3 (1)
Mean (SD) providers seen	1.2 (0.4)	1 (0)	1.3 (0.6)	1 (0)	1 (0)	1.4 (0.8)	1.4 (0.7)	1 (0.2)	1.5 (0.6)	1 (0.1)	1 (0)	1 (0.1)
Top 3 services provided												
1	Child health	Child health	Child health	Child health	Child health	Child health	Child health	Child health	Child health	Child health	Child health	Child health
2	FP counselling	ANC	HIV Treatment	ANC	ANC	ANC	ANC	HIV counselling	FP counselling	FP counselling	ANC	FP counselling
3	FP provision	FP provision	ANC	FP provision	FP provision	FP provision	FP provision	FP counselling	ANC	FP provision	FP provision	PNC
Setting (urban/rural)	Urban	Rural	Rural	Rural	Rural	Rural	Urban	Rural	Rural	Rural	Rural	Rural

HIV-FP model	District C1 facilities (visits n=1641)						District C2 facilities (visits n=1073)					
	a	b	c	d	e	f	g	h	i	j	k	l
Visits tracked	326	318	190	277	250	280	191	218	139	126	230	169
Client category												
Adult (12+ years) only, %	101 (31)	147 (46.2)	69 (36.3)	123 (44.4)	117 (46.8)	159 (56.8)	64 (33.5)	97 (44.5)	56 (40.3%)	45 (35.7)	119 (51.7)	45 (26.6)
Adult+child (%)	225 (69)	171 (53.8)	121 (63.7)	154 (55.6)	133 (53.2)	121 (43.2)	127 (66.5)	121 (55.5)	83 (59.7)	81 (64.3)	111 (48.3)	124 (73.4)
Client gender												
Male (%)	1 (0.3)	0	0	0	46 (18.4)	42 (15)	0	0	0	0	4 (1.7)	0
Female (%)	325 (99.7)	318 (100)	190 (100)	277 (100)	204 (81.6)	237 (84.6)	191 (100)	218 (100)	139 (100)	126 (100)	226 (98.3)	169 (100)
Client age												
Mean (SD)	26.2 (5.7)	26.5 (6.2)	24.7 (4.9)	30.7 (12.1)	30.4 (13.6)	33.8 (14.2)	27.4 (6.8)	27.4 (6.8)	26.7 (6.6)	25.7 (6.4)	28.7 (10.6)	27.3 (6.5)
Services received per visit												
None (%)	0	0	2 (1.1)	8 (2.9)	0	2 (0.7)	0	2 (0.9)	1 (0.7)	13 (10.3)	2 (0.9)	0
One (%)	120 (36.8)	107 (33.6)	73 (38.4)	123 (44.4)	151 (60.4)	69 (24.6)	73 (38.2)	21 (9.6)	61 (43.9)	53 (42.1)	127 (55.2)	73 (43.2)
Two or more (%)	206 (63.2)	211 (66.4)	115 (60.5)	146 (52.7)	99 (39.6)	209 (74.6)	118 (61.8)	195 (89.4)	77 (55.4)	60 (47.6)	101 (43.9)	96 (56.8)
Visits in which ≥1 service received												
Mean (SD) services received	2.1 (1.3)	2.4 (1.5)	1.9 (0.9)	1.8 (0.9)	1.6 (0.9)	2.3 (1.3)	1.9 (0.9)	2.9 (1.3)	1.9 (1)	1.6 (0.6)	1.7 (1)	1.8 (1)
Mean (SD) providers seen	2 (1)	1.2 (0.5)	1.1 (0.4)	1.2 (0.4)	1.1 (0.3)	1.7 (0.8)	1.2 (0.5)	2 (0.7)	1.3 (0.5)	1 (0)	1 (0.2)	1.1 (0.3)
Top 3 services provided												
1	Child health	Child health	Child health	Child health	Other services	Other services	Child health	Child health	Child health	Child health	Child health	Child health
2	ANC	ANC	ANC	FP provision	Child health	Pharmacy	ANC	ANC	ANC	ANC	FP provision	ANC
3	FP provision	FP counselling	FP counselling	FP counselling	FP counselling	Child welfare	FP provision	Other services	FP provision	FP provision	ANC	FP provision
Setting (urban/rural)	Urban	Urban	Urban	Rural	Rural	Rural	Urban	Urban	Rural	Rural	Rural	Rural

ANC, antenatal care; FP, family planning; PNC, postnatal care.

**Figure 1:**
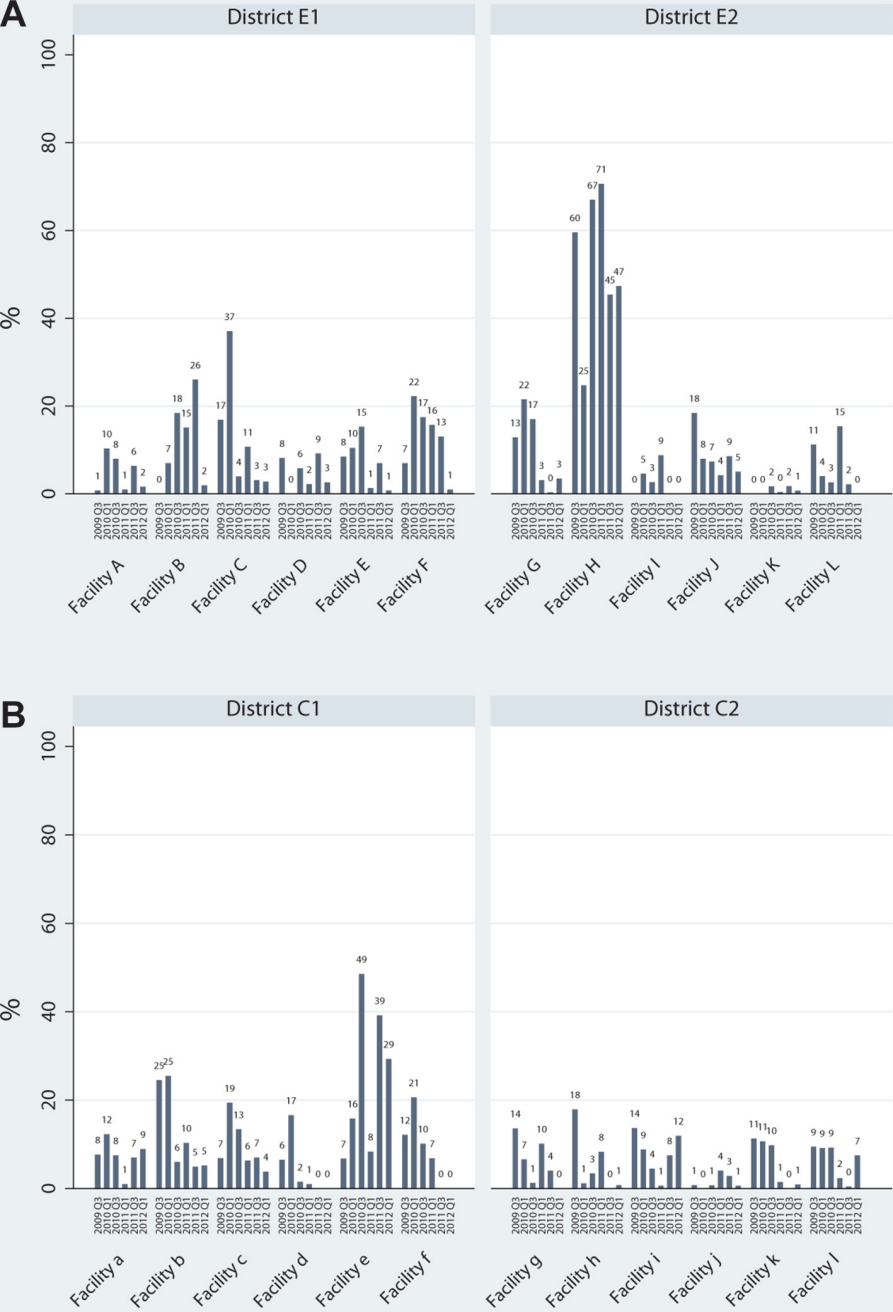
Proportion of visits in which integrated HIV-RH services were received, by facility and round for HIV-PNC model (A) and HIV-FP model (B). FP, family planning; PNC, postnatal care; RH, reproductive health.

In terms of the main outcome—the proportion of visits in which integrated HIV-RH services were received—a minority of clients received integrated services in all facilities and at almost every time point (figure 1A,B). There was one exception in each of the study sites. In the HIV-PNC model districts integrated visits were high in Facility H (even from baseline) and reached 71% in 2011. In the HIV-FP model sites, Facility ‘e’ also stood out, with integrated services received in 49% of visits in one round. In most other cases, integrated services were received at very low levels or not at all.

In the six facilities that received Integra’s HIV-PNC intervention (A–F), baseline levels of integration ranged from 0% to 17%, and rose by the first follow-up round in four facilities (table 2A). Integration remained higher than baseline through subsequent rounds in three sites; however, by the final round, integration levels were lower or unchanged from baseline levels in all sites. Among the six facilities (G–L) that did not receive the Integra HIV-PNC intervention in Eastern Province, integration ranged from 0% to 60% at baseline, increased in two sites and fell or remained unchanged in four sites by the first follow-up. Almost all facilities experienced a decline over subsequent rounds, and by the final round, integration was lower or unchanged from baseline levels in four and two sites, respectively.

**Table 2:** Percentage change in primary outcome (integrated HIV-RH services) and secondary outcome (HIV counselling) over time since baseline

(A) HIV-PNC model												
District E1							District E2					
HIV-RH integration	Facility A	Facility B	Facility C	Facility D	Facility E	Facility F	Facility G	Facility H	Facility I	Facility J	Facility K	Facility L
2010 Q1	9.5 (5.2, 13.9)	7.0 (2, 12)	20.2 (7.1, 33.3)	−8.1 (−12.8, −3.5)	2.0 (−5.5, 9.4)	15.2 (6.2, 24.3)	8.6 (1.1, 16.1)	−34.0 (−43.7, −26)	4.6 (1.3, 7.9)	−10.0 (−19.8, −1)	0	−7.2 (−14.1, −0.3)
2010 Q3	7.2 (3.3, 11)	18.4 (9.7, 27.1)	−12.0 (−21.8, −4)	−2.3 (−8.8, 4.1)	6.8 (−1.4, 14.9)	10.4 (1, 19.9)	4.1 (−2.5, 10.8)	7.4 (−3.5, 18.3)	2.7 (−0.3, 5.7)	−11.0 (−20.6, −1.6)	1.7 (−0.6, 4.1)	−8.6 (−16.4, −0.7)
2011 Q1	0.2 (−1.2, 1.7)	15.1 (8.3, 21.9)	−6.1 (−17.4, 5.1)	−5.9 (−11.2, −0.7)	−7.2 (−12.8, −1.6)	8.7 (0.9, 16.5)	−9.8 (−14.7, −4.8)	11.0 (2.1, 20)	8.8 (3.8, 13.8)	−14.0 (−23.2, −5)	0.4 (−0.4, 1.2)	4.2 (−7.3, 15.7)
2011 Q3	5.6 (2.8, 8.4)	26.0 (19.5, 32.5)	−13.0 (−22.6, −4.9)	1.0 (−6.1, 8.1)	−1.5 (−8.8, 5.8)	6.0 (−3.3, 15.4)	−12.0 (−17.2, −7.9)	−14.0 (−24.8, −3.6)	0	−9.8 (−20.3, 0.6)	1.7 (−0.2, 3.7)	−9.0 (−16.4, −1.7)
2012 Q1	0.9 (−0.9, 2.7)	1.9 (−0.7, 4.6)	−14.0 (−22.7, −5.4)	−5.5 (−11.4, 0.3)	−7.7 (−13.2, −2.2)	−6.0 (−11.4, −0.6)	−9.4 (−14.4, −4.3)	−12.0 (−22.9, −1.6)	0	−13.0 (−22.8, −3.9)	0.7 (−0.7, 2.1)	−11.0 (−17.2, − 5.2)

HIV counselling	Facility A	Facility B	Facility C	Facility D	Facility E	Facility F	Facility G	Facility H	Facility I	Facility J	Facility K	Facility L
2010 Q1	9.0 (4.8, 13.3)	6.0 (1.3, 10.7)	21.9 (10.2, 33.6)	−5.2 (−9.4, −0.9)	2.9 (−4.3, 10.1)	16.4 (7.8, 24.9)	7.6 (0.1, 15.2)	−30 (−39.8, −21.9)	3.9 (0.8, 7)	−3.4 (−12.4, 5.6)	0	−7.2 (−14.1, −0.3)
2010 Q3	6.7 (3, 10.4)	7.9 (1.8, 14)	−5.0 (−12.4, 2.3)	−2.0 (−7.5, 3.4)	8.5 (0.4, 16.5)	12.4 (3.4, 21.5)	4.4 (−2.4, 11.3)	5.2 (−6.1, 16.4)	1.8 (−0.7, 4.2)	−8.3 (−16.7, 0.1)	1.7 (−0.6, 4.1)	−11 (−17.2, − 5.2)
2011 Q1	−0.1 (−1.4, 1.2)	15.1 (8.3, 21.9)	1.7 (−8.3, 11.8)	−5.9 (−9.9, −1.9)	−5.6 (−11.1, −0.1)	10.7 (3.3, 18.1)	−11.0 (−16.5, −6.5)	17.5 (8.7, 26.4)	7.2 (2.7, 11.7)	−9.5 (−17.9, −1.2)	0	6.1 (−5.8, 18)
2011 Q3	8.4 (5.1, 11.7)	30.6 (23.8, 37.5)	−9.0 (−14.9, −3)	−5.9 (−9.9, −1.9)	5.5 (−2.8, 13.7)	8.0 (−1, 17.1)	−13.0 (−18.6, −9.1)	−6.2 (−16.9, 4.5)	0	−13 (−21, −6.5)	6.4 (2.7, 10.1)	−9.0 (−16.4, −1.7)
2012 Q1	4.1 (1.3, 6.9)	0	−7.6 (−14.1, − 1.1)	−5.9 (−9.9, −1.9)	−7.5 (−12.6, −2.5)	−4.0 (−8.7, 0. 7)	−10.0 (−15.8, −5.6)	−11 (−22.5, −1.3)	0	−10 (−18.4, −1.6)	0.7 (−0.7, 2.1)	−11 (−17.2, −5.2)

(B) HIV-FP model												
District C1							District C2					
HIV-RH integration	Facility a	Facility b	Facility c	Facility d	Facility e	Facility f	Facility g	Facility h	Facility i	Facility j	Facility k	Facility l
2010 Q1	4.6 (−0.5, 9.7)	1.0 (−6.2, 8.1)	12.6 (6.3, 18.8)	10.1 (4.5, 15.7)	9.0 (1.8, 16.3)	8.5 (0.5, 16.5)	−7.0 (−12.7, −1.3)	−16.0 (−22, −11.4)	−4.8 (−13.7, 4)	−0.8 (−2.3, 0.8)	−0.6 (−7.3, 6.1)	−0.3 (−6.7, 6)
2010 Q3	−0.2 (−4.4, 4.1)	−18.0 (−24.1, −13)	6.6 (0.8, 12.3)	−5.0 (−8.3, −1.6)	41.7 (31.6, 51.9)	−2.0 (−8.3, 4.3)	−12.0 (−17.4, −7.3)	−14.0 (−20.1, −8.9)	−9.2 (−15.6, −2.7)	0 (−2.2, 2.1)	−1.5 (−7.9, 4.8)	−0.2 (−6.5, 6.1)
2011 Q1	−6.7 (−9.7, −3.6)	−14.0 (−20, −8.4)	−0.5 (−5.1, 4.1)	−5.5 (−8.7, −2.3)	1.5 (−4.5, 7.6)	−5.3 (−11.3, 0.7)	−3.5 (−10.5, 3.5)	−9.6 (−15.7, −3.5)	−13.0 (−18.9, −7.2)	3.3 (0.1, 6.4)	−9.8 (−14.4, −5.3)	−7.1 (−12.1, −2.2)
2011 Q3	−0.7 (−4.6, 3.2)	−19.0 (−24.9, −14.3)	0.2 (−4.6, 4.9)	−6.5 (−9.4, −3.6)	32.4 (20.8, 43.9)	−12.0 (−16, −8.3)	−9.6 (−14.8, −4.3)	−17.0 (−23, −12.8)	−6.2 (−13.6, 1.2)	2.0 (−0.7, 4.8)	−11.0 (−15.4, −7.2)	−9.0 (−13.5, −4.5)
2012 Q1	1.3 (−2.4, 5)	− 19.0	−3.0 (−7.1, 1.1)	−6.5 (−9.4, −3.6)	22.5 (10.4, 34.6)	−12.0 (−16, −8.3)	−13.0 (−18.5, −8.7)	−17.0 (−22.3, −11.9)	−1.7 (−9.7, 6.2)	0.2 (−2.1, 1.8)	−10.0 (−14.7, −6.1)	−2.0 (−8.6, 4.7)
		(−24.6, −14)										

HIV counselling	Facility a	Facility b	Facility c	Facility d	Facility e	Facility f	Facility g	Facility h	Facility i	Facility j	Facility k	Facility l
2010 Q1	13.7 (9, 19.3)	−1.0 (−7.8, 5.8)	14.6 (7.6, 21.6)	3.0 (−1.2, 7.2)	8.0 (2.1, 14)	7.1 (−1.1, 15.4)	−5.6 (−11.2, 0.1)	−16.0 (−21.3, − 11.1)	−2.7 (−11.3, 5.9)	−0.8 (−2.3, 0.8)	−2.8 (−9.7, 4.1)	−0.4 (−6.5, 5.8)
2010 Q3	0.6 (−3.2, 4.4)	−20.0 (−25.6, −15.7)	4.3 (−2, 10.5)	−4.2 (−6.9, −1.5)	52.5 (42.7, 62.4)	−0.5 (−7.6, 6.5)	−7.6 (−13, −2.1)	−14.0 (−19.4, −8.6)	−6.5 (−12.7, −2)	−0.8 (−2.3, 0.8)	−1.6 (−8.5, 5.3)	−0.9 (−6.8, 5.1)
2011 Q1	−5.8 (−8.4, −3.3)	−12.0 (−17.7, −6.3)	−4.0 (−9.2, 1.1)	−4.7 (−7.2, −2.2)	0.9 (−3.2, 5)	−7.4 (−13.6, −1.3)	−4.6 (−11.1, 1.9)	−9.0 (−15, −3.1)	−10.0 (−16.3, −5.4)	−0.3 (−2.1, 1.6)	−12.0 (−17.4, −8.1)	−8.3 (−12.7, −3.9)
2011 Q3	−0.8 (−4.2, 2.6)	−19.0 (−24.1, −14)	−3.8 (−9, 1.4)	−4.1 (−6.9, −1.4)	49.9 (38.3, 61.5)	−8.7 (−14.5, −3)	−4.8 (−10.2, 0.7)	−17.0 (−22, −12)	−3.2 (−10.4, 4.1)	0.6 (−1.6, 2.8)	−12.0 (−17.4, −8.1)	−7.9 (−12.4, −3.4)
2012 Q1	2.3 (−1.1, 5.6)	−17.0 (−22.6, −12,3)	−5.9 (−10.6, −1.2)	−4.7 (−7.2, −2.2)	21.3 (10.1, 32.5)	−14.0 (−18.4, −10.2)	−12.0 (−17.3, −7.9)	−17.0 (−22, −12)	0.4 (−7.2, 8.1)	−0.8 (−2.3, 0.8)	−12.0 (−17.1, −8)	−2.3 (−8.7, 4)

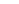
Evidence of increase from baseline level.

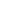
Evidence of decline from baseline level.

FP, family planning; PNC, postnatal care; RH, reproductive health.

In the six facilities receiving the HIV-FP intervention (Facilities a–f), integration ranged from 6% to 23% at baseline. As also observed in the HIV-PNC intervention sites, integration rose significantly by the first follow-up round in four facilities (table 2B); levels remained unchanged in the other two sites. In almost all sites, integration peaked at the first follow-up and subsequently dropped below baseline levels at almost all follow-ups, in some cases steadily and other cases dramatically. While integration also declined over time in Facility ‘e’, levels remained higher than all other facilities in Central Province, and significantly higher than initial levels by the final round (29% vs 7% at baseline). Among the six facilities that did not receive the HIV-FP intervention, levels of integration were generally low and showed no increase at first follow-up and almost all other rounds.

**Figure 2:**
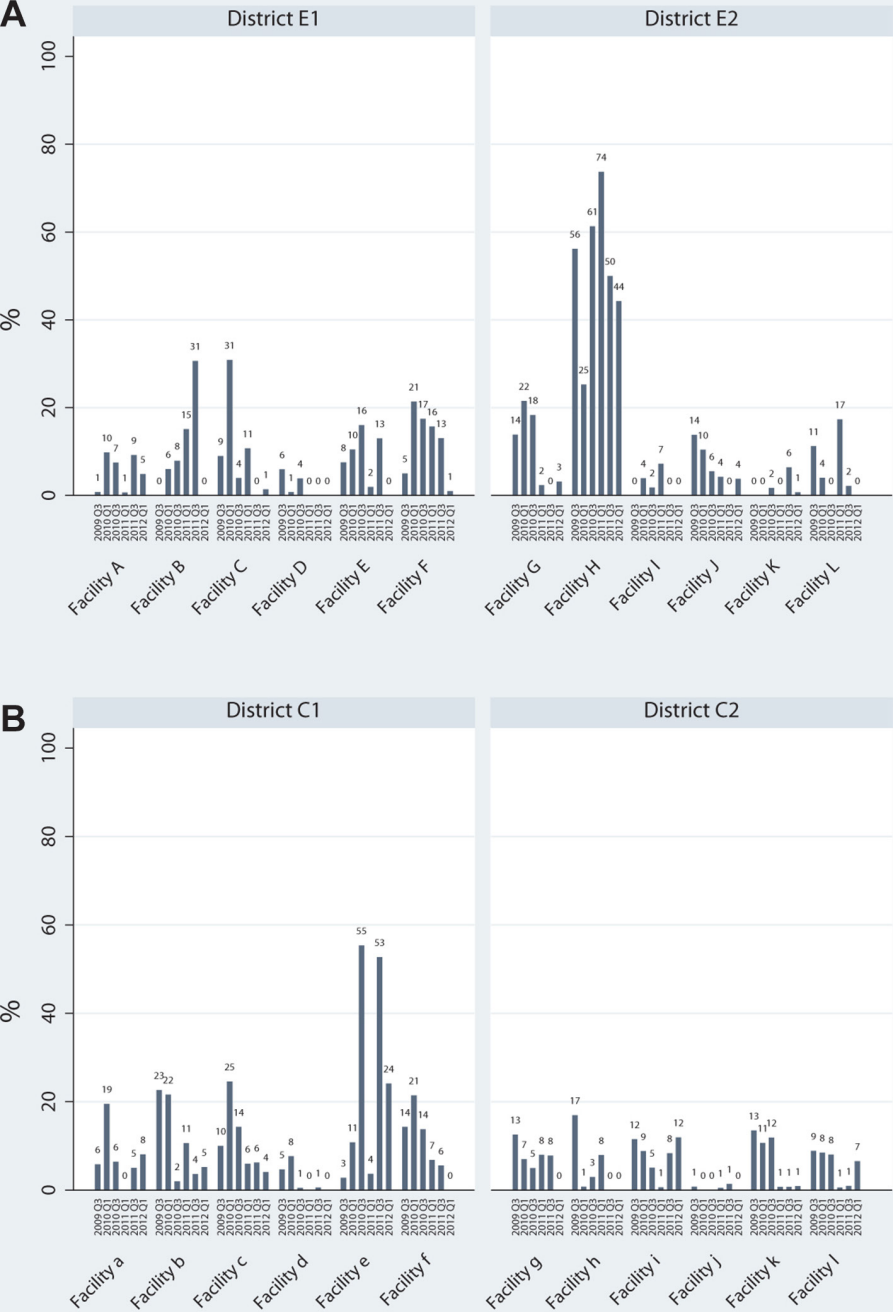
Proportion of visits in which HIV counselling was received, by facility and round for HIV-PNC model (A) and HIV-FP model (B). FP, family planning; PNC, postnatal care.

By facility, receipt of HIV counselling, presented in figure 2A,B, showed a similar pattern to integrated services described above. In the HIV-PNC model sites, levels of HIV counselling rose significantly by the first follow-up round in the same six facilities that experienced rises in HIV-RH integration (table 2A). Levels at subsequent follow-up rounds frequently mirrored the changes observed in integration. In the HIV-FP study sites, levels and changes in HIV counselling also closely matched those of integration (table 2B), with an immediate rise at first follow-up in almost all ‘intervention’ sites. Across facilities, HIV counselling varied considerably, with many facilities providing no HIV counselling or low levels throughout the study. In an exception, a majority of clients in Facility H received HIV testing in most rounds, reaching a maximum of 74% at one point. In Central Province, Facility ‘e’ showed much higher levels than all other facilities, reaching 55%. HIV counselling was generally very low among ‘comparison’ sites. In both provinces, across all sites, all but one facility showed no increase in individual HIV counselling by the final round compared with baseline levels.

**Figure 3:**
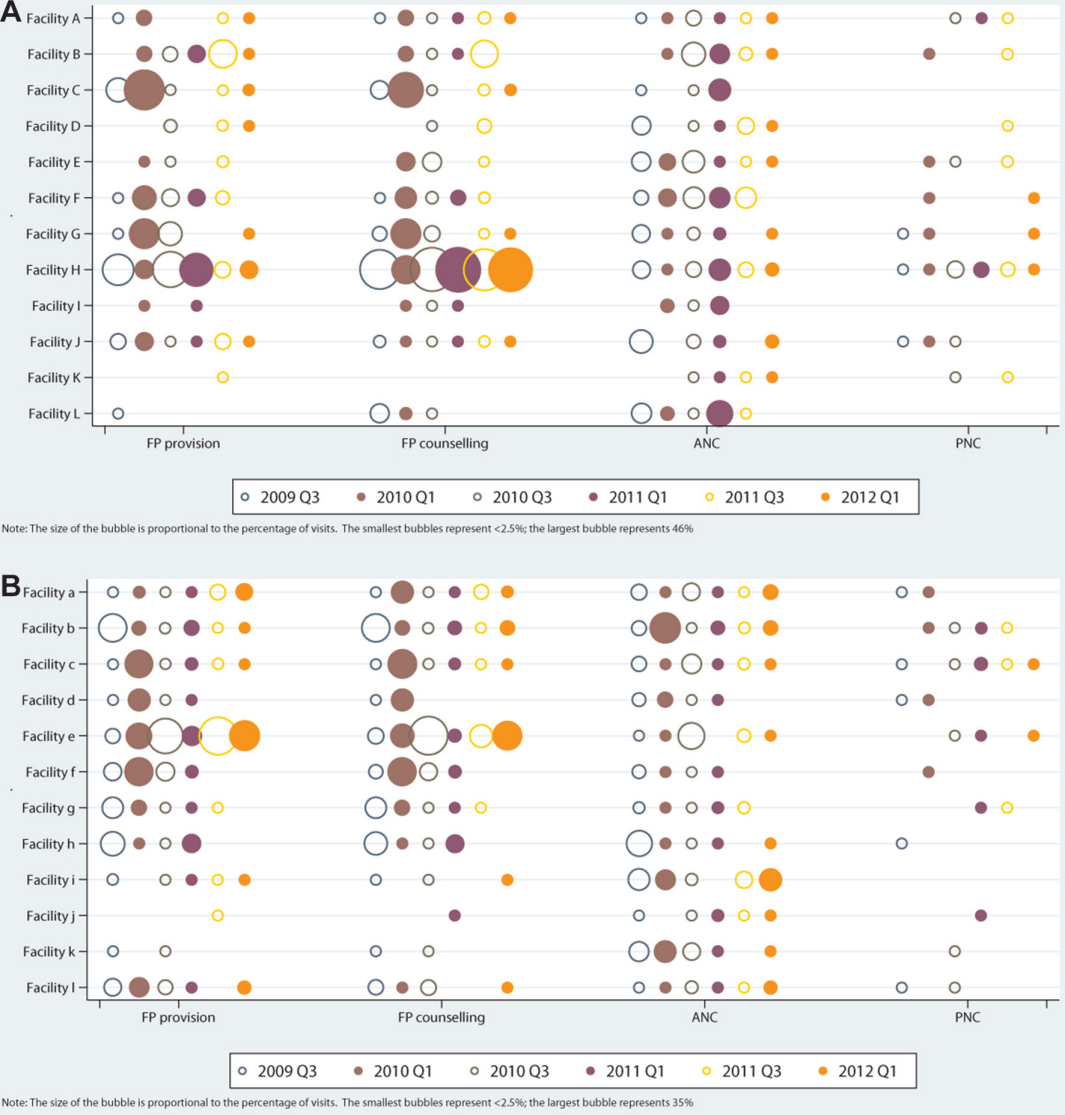
Proportion of RH visits in which an HIV/STI service was also received, by facility, RH service and round for HIV-PNC integration model (A) and HIV-FP model (B). ANC, antenatal care; FP, family planning; PNC, postnatal care; RH, reproductive health; STI, sexually transmitted infection.

Figure 3A,B shows the combinations of RH services that were received with HIV/STI services. The most consistent combination—across sites and rounds—was integration of HIV/STI with ANC. Integration was also common with FP provision and counselling—particularly in Facilities ‘H’ and ‘e’ which showed exceptionally high levels of overall integration. In the first follow-up round, when most ‘intervention’ sites saw a rise in overall integration, combinations of HIV and FP services were at their highest levels for most sites. In general, HIV/STI services were least often received with PNC.

## Discussion

The collection and assessment of 25 539 client visits over six time points enabled a rare, detailed picture of HIV-RH integration in government facilities in Africa. Tracking the flow of clients throughout their consultation allowed us to document combinations of services received, with a level of detail typically unavailable from routine health information systems. The data showed significant heterogeneity across facilities, yet a consistent pattern emerged: there were initial, short-term effects of the Integra Intervention at facility level evidenced by a rise in integration at the first follow-up round in most intervention sites. These were followed by declines in both HIV-RH integration and HIV counselling (the secondary outcome) across all four districts in all but a few individual clinics.

Where integration occurred it was largely driven by rises in HIV counselling, which appears to act as the ‘glue’ or linking service with core RH services. On the RH side, the most consistent combination was between HIV services and ANC. This predated the Integra Initiative (often through widespread PMTCT initiatives funded by the US Government) and remained evident in most facilities—both intervention and comparison—by the final round. These findings are consistent with the patterns of integration documented via CFAs in Swaziland,^22^ Kenya and India,^27^ in which child health, ANC and FP offered the best opportunities for integration with HIV, and HIV-PNC integration was observed least often. The collection of data at six time points in this analysis, versus only three time points in Swaziland (and one time point in the cross-sectional study conducted in India and Kenya in 2014^27^) allowed for more detailed observations over time, including the initial, short-term boost that would have been missed in Swaziland.

The initial boost in integration at the first follow-up round in Kenya seemed to be driven by linkages with FP counselling and provision. HIV-FP integration was also the most common combination in the two sites that delivered the highest levels of integration throughout the study (Facilities ‘H’ and ‘e’). Thus, all time points when integration was highest were due to HIV-FP integration, suggesting that FP has an important role to play in scaling up integration.

Unlike HIV-ANC integration, which remained consistent, levels of HIV-FP integration were not sustained across the study period. This reflects the challenge of institutionalising an initiative like integration, without continued funding and prioritisation as with PMTCT which has attracted substantial and sustained funding from large donors as part of the fight against HIV transmission to children. For routine testing in FP services, the supply of HIV testing kits, without which providers are unable to provide testing services and are less likely to offer HIV counselling, is also important. Related qualitative work also highlights the importance of providers. Provider commitment was challenged by staffing shortages, rotations and turnovers and increased workload. The importance of supervisory support, teamwork, staff being able to make flexible decisions and good communications between providers and across clinics within facilities—in the delivery of integrated care—has been observed in related publications.^19 28^

Further work—case studies by the Integra Initiative—has analysed drivers of integration and sought to explain and learn from the heterogeneity observed across facilities, for example, the facilities which showed the highest levels of integration (like H and e), and the lowest (A, K J), and those which experienced steady declines (Facilities F and f) or rises (B) or the steepest drops (C, b). Considerable variation across facilities was also observed in CFAs in India and Kenya.^27^ The Integra case study analyses^12^ draw on multiple methods, including qualitative and context data to explore the interactions of structural factors and ‘people’ factors (also called ‘health systems software’) that influence integrated service delivery. Findings from the case studies suggest that although structural factors like stock-outs, distribution of staffing and workload, and rotation of staff can affect how integrated care is provided, all these factors can be influenced by staff themselves: both front line and management. The high-performing Facility H (reported in the related paper as Clinic 1) and the dramatically improving Facility ‘e’ (reported as Clinic 14) had staff who displayed agency of decision-making, worked as a team to share workload and had management that supported this. As a result, staff were able to overcome some structural deficiencies to enable integrated care, despite challenges. Conversely, the steadily declining Facility ‘f’ (‘Clinic 2’ and others) showed that despite good structural integration staff were unable to use this because they were poorly organised, unsupported or teams were dysfunctional. Conscientious objection and moralistic attitudes were also barriers. Overall, the case studies show that the integration of structural components (like buildings and clinical supplies) is insufficient to achieve integrated service delivery; rather, integration relies on the commitment, consistency and agency of individual providers and teams. Excellent management leadership and teamwork of front-line providers can ensure facilities perform well despite structural challenges.^12^ The insights form the case studies also indicate that to meaningfully interpret large, complex data sets for an intervention as nuanced and diverse as ‘service integration’, qualitative, process and contextual data are needed.

There are a number of recommendations that arise from this study. First, the finding that HIV counselling appears to act as the linking service with core RH services points to a key entry point for integrated delivery of FP. Integration of HIV counselling and testing services should therefore be rapidly scaled up within FP services in contexts of generalised HIV epidemics. Second, the fact that the short-term boost in integration that was observed in almost all intervention sites (driven by integration of HIV and FP services) was not sustained, underlines the need for continued funding and prioritisation. Findings also showed that integration happens most consistently between HIV services and ANC which directly reflects the widespread and sustained support for PMTCT initiatives. Just as PMTCT services have enjoyed long-term funding and prioritisation, this same attention must now be given by donors and implementing agencies to integrating HIV counselling and testing within FP services. Finally, the wider Integra analyses indicate that to meaningfully interpret large, complex data sets like those presented in this paper, mixed-methods approaches are needed to provide qualitative and process-level insights that explain the patterns in big data sets.

This study has provided a rare snapshot of detailed client-level time-series data on clients’ receipt of integrated services in four districts in Kenya, allowing us to better understand the patterns of integrated service provision as well as the magnitude of the task that remains to sustain integrated service delivery in public sector facilities.

10.1136/bmjgh-2018-000867.supp4 Supplementary file 4

10.1136/bmjgh-2018-000867.supp5 Supplementary file 5
