# Supplementary material for: Integration of HIV and reproductive health services in public sector facilities: analysis of client flow data over time in Kenya
Source: BMJ Glob Health. 2018 Sep 14;3(5):e000867. doi: 10.1136/bmjgh-2018-000867 (PMC6144905; doi:10.1136/bmjgh-2018-000867)
Supplement: Supplementary file 1 [file bmjgh-2018-000867supp001.pdf]

KENYA CLIENT FLOW FORM

Client No: ☐☐/☐☐☐

Facility name: ________________________ **Entry unit:** MCH ☐ Other: _____________________

Date today: ☐☐-☐☐-☐☐ **Client arrival time:** ☐☐:☐☐ **Sex of client:** ☐Male ☐Female

Age: ☐☐ **DOB:** ☐☐-☐☐-☐☐ **Client type:** ☐ Adult (alone) ☐ Adult with child (age of child_____)

Client's residence: District: _________________ Division: ________________ Location: ______________

Instructions to client: Please take this form to each nurse, doctor or other counselor that you see during your visit at this clinic today. Please hand the form back in to one of the interviewers before you leave.

Instructions to the provider: Please complete a new row for each client you see. Fill in the time that the client arrives, and the time the client leaves. In the first column, tick for each of the services that you provide to the client. If you refer the client somewhere else, tick the appropriate box(es) in the second column, and indicate if it was an internal or external referral (or both). If you do not refer the client on, leave question 2 blank.

1st Provider seen:

Consultation start time: ☐☐:☐☐ **Consultation end time:** ☐☐:☐☐

1. What is client seen for? (tick all that apply)

- ☐ 1.Ante-natal care
- ☐ 2.Child immunization
- ☐ 3.Child welfare
- ☐ 4.Counseling (general)
- ☐ 5.Family planning counseling
- ☐ 6.Family planning provision
- ☐ 7.Gynecologist
- ☐ 8.HIV care (pre ART or ART)
- ☐ 9.HIV counseling
- ☐ 10.HIV testing (blood test)
- ☐ 11.Laboratory test
- ☐ 12.Pap smear
- ☐ 13.Pharmacy (drugs)
- ☐ 14.PMTCT
- ☐ 15.PNC baby (exam)
- ☐ 16.PNC mother (exam)
- ☐ 17.STI counseling
- ☐ 18.STI treatment
- ☐ 19.TB care/treatment
- ☐ 20.X-ray
- ☐ 21.Other (specify): ______________________

2. What is client referred for? (tick all that apply)

- ☐ Ante-natal care
- ☐ Child immunization
- ☐ Child welfare
- ☐ Counseling (general)
- ☐ Family planning counseling
- ☐ Family planning provision
- ☐ Gynecologist
- ☐ HIV care (pre ART or ART)
- ☐ HIV counseling
- ☐ HIV testing (blood test)
- ☐ Laboratory test
- ☐ Pap smear
- ☐ Pharmacy (drugs)
- ☐ PMTCT
- ☐ PNC for baby (exam)
- ☐ PNC for mother (exam)
- ☐ STI counseling
- ☐ STI treatment
- ☐ TB care/treatment
- ☐ X-ray
- ☐ Other (specify): ______________________

Is this/are these: ☐Internal referral(s) ☐External referral(s)
If external, where? __________________________________

2nd Provider seen:

Consultation start time: ☐☐:☐☐ **Consultation end time:** ☐☐:☐☐

1. What is client seen for? (tick all that apply)

- ☐ Ante-natal care
- ☐ Child immunization
- ☐ Child welfare
- ☐ Counseling (general)
- ☐ Family planning counseling
- ☐ Family planning provision
- ☐ Gynecologist
- ☐ HIV care (pre ART or ART)
- ☐ HIV counseling
- ☐ HIV testing (blood test)
- ☐ Laboratory test
- ☐ Pap smear
- ☐ Pharmacy (drugs)
- ☐ PMTCT
- ☐ PNC for baby (exam)
- ☐ PNC for mother (exam)
- ☐ STI counseling
- ☐ STI treatment
- ☐ TB care/treatment
- ☐ X-ray
- ☐ Other (specify): ______________________

2. What is client referred for? (tick all that apply)

- ☐ Ante-natal care
- ☐ Child immunization
- ☐ Child welfare
- ☐ Counseling (general)
- ☐ Family planning counseling
- ☐ Family planning provision
- ☐ Gynecologist
- ☐ HIV care (pre ART or ART)
- ☐ HIV counseling
- ☐ HIV testing (blood test)
- ☐ Laboratory test
- ☐ Pap smear
- ☐ Pharmacy (drugs)
- ☐ PMTCT
- ☐ PNC for baby (exam)
- ☐ PNC for mother (exam)
- ☐ STI counseling
- ☐ STI treatment
- ☐ TB care/treatment
- ☐ X-ray
- ☐ Other (specify): ______________________

Is this/are these: ☐Internal referral(s) ☐External referral(s)
If external, where? __________________________________

3rd Provider seen: **Consultation start time:** ☐☐:☐☐ **Consultation end time:** ☐☐:☐☐

1. What is client seen for? (tick all that apply)

☐ Ante-natal care	☐ Pap smear
☐ Child immunization	☐ Pharmacy (drugs)
☐ Child welfare	☐ PMTCT
☐ Counseling (general)	☐ PNC for baby (exam)
☐ Family planning counseling	☐ PNC for mother (exam)
☐ Family planning provision	☐ STI counseling
☐ Gynecologist	☐ STI treatment
☐ HIV care (pre ART or ART)	☐ TB care/treatment
☐ HIV counseling	☐ X-ray
☐ HIV testing (blood test)	☐ Other (specify):
☐ Laboratory test	______________________

2. What is client referred for? (tick all that apply)

☐ Ante-natal care	☐ Pap smear
☐ Child immunization	☐ Pharmacy (drugs)
☐ Child welfare	☐ PMTCT
☐ Counseling (general)	☐ PNC for baby (exam)
☐ Family planning counseling	☐ PNC for mother (exam)
☐ Family planning provision	☐ STI counseling
☐ Gynecologist	☐ STI treatment
☐ HIV care (pre ART or ART)	☐ TB care/treatment
☐ HIV counseling	☐ X-ray
☐ HIV testing (blood test)	☐ Other (specify):
☐ Laboratory test	______________________

Is this/are these: ☐ Internal referral(s) ☐ External referral(s)
If external, where? ______________________________

4th Provider seen: **Consultation start time:** ☐☐:☐☐ **Consultation end time:** ☐☐:☐☐

1. What is client seen for? (tick all that apply)

☐ Ante-natal care	☐ Pap smear
☐ Child immunization	☐ Pharmacy (drugs)
☐ Child welfare	☐ PMTCT
☐ Counseling (general)	☐ PNC for baby (exam)
☐ Family planning counseling	☐ PNC for mother (exam)
☐ Family planning provision	☐ STI counseling
☐ Gynecologist	☐ STI treatment
☐ HIV care (pre ART or ART)	☐ TB care/treatment
☐ HIV counseling	☐ X-ray
☐ HIV testing (blood test)	☐ Other (specify):
☐ Laboratory test	______________________

2. What is client referred for? (tick all that apply)

☐ Ante-natal care	☐ Pap smear
☐ Child immunization	☐ Pharmacy (drugs)
☐ Child welfare	☐ PMTCT
☐ Counseling (general)	☐ PNC for baby (exam)
☐ Family planning counseling	☐ PNC for mother (exam)
☐ Family planning provision	☐ STI counseling
☐ Gynecologist	☐ STI treatment
☐ HIV care (pre ART or ART)	☐ TB care/treatment
☐ HIV counseling	☐ X-ray
☐ HIV testing (blood test)	☐ Other (specify):
☐ Laboratory test	______________________

Is this/are these: ☐ Internal referral(s) ☐ External referral(s)
If external, where? ______________________________

5th Provider seen: **Consultation start time:** ☐☐:☐☐ **Consultation end time:** ☐☐:☐☐

1. What is client seen for? (tick all that apply)

☐ Ante-natal care	☐ Pap smear
☐ Child immunization	☐ Pharmacy (drugs)
☐ Child welfare	☐ PMTCT
☐ Counseling (general)	☐ PNC for baby (exam)
☐ Family planning counseling	☐ PNC for mother (exam)
☐ Family planning provision	☐ STI counseling
☐ Gynaecologist	☐ STI treatment
☐ HIV care (pre ART or ART)	☐ TB care/treatment
☐ HIV counseling	☐ X-ray
☐ HIV testing (blood test)	☐ Other (specify):
☐ Laboratory test	______________________

2. What is client referred for? (tick all that apply)

☐ Ante-natal care	☐ Pap smear
☐ Child immunization	☐ Pharmacy (drugs)
☐ Child welfare	☐ PMTCT
☐ Counseling (general)	☐ PNC for baby (exam)
☐ Family planning counseling	☐ PNC for mother (exam)
☐ Family planning provision	☐ STI counseling
☐ Gynecologist	☐ STI treatment
☐ HIV care (pre ART or ART)	☐ TB care/treatment
☐ HIV counseling	☐ X-ray
☐ HIV testing (blood test)	☐ Other (specify):
☐ Laboratory test	______________________

Is this/are these: ☐ Internal referral(s) ☐ External referral(s)
If external, where? ______________________________
