# Supplementary material for: Integration of HIV and reproductive health services in public sector facilities: analysis of client flow data over time in Kenya
Source: BMJ Glob Health. 2018 Sep 14;3(5):e000867. doi: 10.1136/bmjgh-2018-000867 (PMC6144905; doi:10.1136/bmjgh-2018-000867)
Supplement: Supplementary file 2 [file bmjgh-2018-000867supp002.pdf]

Supplemental Table 1. Dates of data collection in Eastern province (HIV-PNC model)

KEY
- Mondays
- Date included in analyses
- Date omitted from analyses

Round 1 (2009 Q	unknown	06 Jul	07 Jul	08 Jul	09 Jul	10 Jul	13 Jul	14 Jul	15 Jul	16 Jul	17 Jul
Facility A		82	72	86	92	58					
Facility B		51	14	12	58	74					
Facility C		8	18	24	19	20					
Facility D		55	9	8	53	10					
Facility E		20	45	15	21	5					
Facility F		14	19	17	23	27					
Facility G							45	29	63	26	39
Facility H							45	67	42	38	43
Facility I							21	29	31	15	19
Facility J							18	24	14	14	17
Facility K							31	54	51	24	61
Facility L							16	8	49	15	19
unknown		1									

Round 2 (2010 Q	unknown	26apr1980	22jan1990	25oct2001	05 Jan	18 Jan	19 Jan	20 Jan	21 Jan	22 Jan	25 Jan	26 Jan	27 Jan	28 Jan	29 Jan	29Jan2016
Facility A		1									35	47	26	51	35	
Facility B						47	3	3	5	42						
Facility C						21	25	13	16	6						
Facility D				1	1						42	28	8	46	11	
Facility E											22	45	14	27	26	
Facility F			1			33	20	12	23	29						
Facility G	1					48	34	55	16	33						
Facility H											34	44	34	38	32	
Facility I						14	32	36	29	42						
Facility J											25	26	28	24	22	
Facility K											43	18	28	14	43	1
Facility L						29	24	30	23	19						

Round 3 (2010 Q	unknown	21 Jun	22 Jun	23 Jun	24 Jun	25 Jun	26 Jun	27 Jun	28 Jun	29 Jun	30 Jun	01 Jul	02 Jul	05 Jul	06 Jul	07 Jul	08 Jul	09 Jul
Facility A		29	49	45	52	26												
Facility B		24	6	7	4	35												
Facility C		4	26	16	30													
Facility D									35	12	6	24	26					
Facility E									45	25	30	23	8					
Facility F	1	34	17	15	10	10												
Facility G								1	65	32	45	39	54					
Facility H									19	41	10	17	19					
Facility I		31	15	23	20	23												
Facility J									29	14	17	18	31					
Facility K									43	23	22	17	12					
Facility L		2	5	14	11	6												

Round 4 (2011 Q	unknown	24 Jan	25 Jan	26 Jan	27 Jan	28 Jan	31 Jan	01 Feb	02 Feb	03 Feb	04 Feb	07 Feb	08 Feb	09 Feb	10 Feb	11 Feb
Facility A		108	45	47	63	36	1									
Facility B		13	8	10	9	66										
Facility C								31		24	1					
Facility D	1	66	3	6	37	23										
Facility E							49	29	11							
Facility F		27	26	13	18	56										
Facility G							72	97	57	69	91					
Facility H		38	47	28	32	49										
Facility I							48	22	18	18	19					
Facility J							12	17	19	24	22					
Facility K												50	57	26	56	58
Facility L							10	8	13	6	15					

Round 5 (2011 Q	unknown	01 Aug	02 Aug	03 Aug	04 Aug	05 Aug	06 Aug	08 Aug	09 Aug	10 Aug	11 Aug	12 Aug	15 Aug	16 Aug	17 Aug	18 Aug	19 Aug
Facility A		50	71	65	77	52											
Facility B	1												56	12	12	21	72
Facility C								1	28	4	24	7					
Facility D		52	8	4	39	6											
Facility E								10	19	20	26	25					
Facility F		18	13	15	14	9											
Facility G								69	51	48	66	67					
Facility H		17	47	20	15	31											
Facility I								27	28	11	8	13					
Facility J		10	8	10	32	10											
Facility K													26	38	20	55	33
Facility L							1	7	17	5	8						

Round 6 (2012 Q	unknown	16 Jan	17 Jan	18 Jan	19 Jan	20 Jan	23 Jan	24 Jan	25 Jan	26 Jan	27 Jan	28 Jan	30 Jan	31 Jan	01 Feb	02 Feb	03 Feb
Facility A													53	52	32	48	61
Facility B		17	24	7	14	42											
Facility C		2	35		31	4											
Facility D													36	6	2	24	8
Facility E							29	43	23	15	24						
Facility F													37	15	7	22	20
Facility G													63	66	64	67	56
Facility H													29	28	42	15	17
Facility I													36	13	11	16	21
Facility J							29	14	18	9	9	1					
Facility K							28	51	17	19	26						
Facility L													22	8	11	11	10
