# Supplementary material for: Integration of HIV and reproductive health services in public sector facilities: analysis of client flow data over time in Kenya
Source: BMJ Glob Health. 2018 Sep 14;3(5):e000867. doi: 10.1136/bmjgh-2018-000867 (PMC6144905; doi:10.1136/bmjgh-2018-000867)
Supplement: Supplementary file 4 [file bmjgh-2018-000867supp004.pdf]

Supplemental Table 3. Characteristics of client visits by facility, round and service type in Eastern Province (HIV-PNC)

District E1 Facilities (Facilities A–F)

	Facility A						Facility B					
	Jun2009	Jan2010	Jun2010	Jan2011	Jun2011	Jan2012	Jun2009	Jan2010	Jun2010	Jan2011	Jun2011	Jan2012
**Visits tracked**	390	194	201	299	315	246	209	100	76	106	173	104
**Client age (years)**												
Mean [sd]	26.7 [7.3]	25.9 [5.8]	26.2 [6]	26.3 [6.4]	26.3 [6.1]	25.9 [5.8]	26.4 [7.5]	27.3 [7.2]	25.5 [5.8]	26.2 [7.4]	26.4 [7.7]	27.3 [7]
Missing	1 (.3%)	3 (1.5%)	1 (.5%)	0 (0%)	2 (.6%)	0 (0%)	0 (0%)	0 (0%)	0 (0%)	0 (0%)	3 (1.7%)	0 (0%)
**Services received per client**												
none	1 (.3%)	0 (0%)	0 (0%)	0 (0%)	0 (0%)	0 (0%)	8 (3.8%)	2 (2%)	0 (0%)	0 (0%)	3 (1.7%)	0 (0%)
one	173 (44.4%)	87 (44.8%)	87 (43.3%)	109 (36.5%)	160 (50.8%)	124 (50.4%)	116 (55.5%)	25 (25%)	42 (55.3%)	31 (29.2%)	73 (42.2%)	65 (62.5%)
two or more	216 (55.4%)	107 (55.2%)	114 (56.7%)	190 (63.5%)	155 (49.2%)	122 (49.6%)	85 (40.7%)	73 (73%)	34 (44.7%)	75 (70.8%)	97 (56.1%)	39 (37.5%)
**Visits where at least one services was received...**												
Mean [sd] services received	1.7 [.8]	1.8 [1]	1.8 [1]	2.2 [1.2]	1.8 [1.1]	1.7 [.9]	1.4 [.5]	2.3 [1.1]	2.1 [1.3]	2.4 [1.4]	2.6 [1.9]	1.6 [1]
Mean [sd] providers seen	1.2 [.4]	1.1 [.5]	1.2 [.4]	1.8 [.8]	1.4 [.6]	1.3 [.5]	1 [0]	1.4 [.6]	1.7 [1]	1.6 [.8]	1.3 [.5]	1.4 [.7]
**Top 3 services provided**												
1	Child welfare	Child welfare	Child welfare	Child welfare	Child welfare	Child welfare	Child welfare	Child welfare	ANC	Child welfare	Child welfare	Child welfare
2	FP counselling	FP provision	FP provision	Other services	Other services	ANC	ANC	ANC	Child welfare	ANC	HIV counselling	ANC
3	FP provision	ANC	FP counselling	pharmacy	FP provision	FP provision	FP provision	FP counselling	FP provision	HIV counselling	FP provision	FP provision
**HIV/STI services received**												
HIV testing	5 (1.3%)	19 (9.8%)	10 (5%)	1 (.3%)	24 (7.6%)	12 (4.9%)	1 (.5%)	7 (7%)	12 (15.8%)	16 (15.1%)	45 (26%)	2 (1.9%)
HIV counselling	3 (.8%)	19 (9.8%)	15 (7.5%)	2 (.7%)	29 (9.2%)	12 (4.9%)	0 (0%)	6 (6%)	6 (7.9%)	16 (15.1%)	53 (30.6%)	0 (0%)
HIV treatment	1 (.3%)	3 (1.5%)	1 (.5%)	0 (0%)	0 (0%)	1 (.4%)	0 (0%)	0 (0%)	1 (1.3%)	1 (.9%)	2 (1.2%)	0 (0%)
HIV PMTCT	0 (0%)	1 (.5%)	4 (2%)	4 (1.3%)	13 (4.1%)	1 (.4%)	0 (0%)	5 (5%)	2 (2.6%)	2 (1.9%)	1 (.6%)	0 (0%)
Other HIV/STI service	0 (0%)	0 (0%)	0 (0%)	0 (0%)	0 (0%)	0 (0%)	0 (0%)	0 (0%)	0 (0%)	0 (0%)	0 (0%)	0 (0%)
STI counselling or testing	0 (0%)	0 (0%)	1 (.5%)	1 (.3%)	1 (.3%)	0 (0%)	0 (0%)	0 (0%)	1 (1.3%)	0 (0%)	1 (.6%)	0 (0%)
Any HIV/STI services	5 (1.3%)	20 (10.3%)	16 (8%)	3 (1%)	33 (10.5%)	12 (4.9%)	1 (.5%)	9 (9%)	14 (18.4%)	17 (16%)	55 (31.8%)	2 (1.9%)
**MCH services received**												
Family Planning Provision	96 (24.6%)	69 (35.6%)	68 (33.8%)	7 (2.3%)	60 (19%)	41 (16.7%)	9 (4.3%)	19 (19%)	14 (18.4%)	9 (8.5%)	34 (19.7%)	19 (18.3%)
Family Planning Counselling	100 (25.6%)	40 (20.6%)	64 (31.8%)	8 (2.7%)	22 (7%)	30 (12.2%)	2 (1%)	19 (19%)	5 (6.6%)	3 (2.8%)	38 (22%)	10 (9.6%)
ANC	65 (16.7%)	56 (28.9%)	42 (20.9%)	48 (16.1%)	39 (12.4%)	61 (24.8%)	56 (26.8%)	24 (24%)	24 (31.6%)	36 (34%)	28 (16.2%)	34 (32.7%)
PNC (mother or baby)	77 (19.7%)	1 (.5%)	5 (2.5%)	15 (5%)	6 (1.9%)	11 (4.5%)	1 (.5%)	5 (5%)	2 (2.6%)	7 (6.6%)	10 (5.8%)	2 (1.9%)
cervical screening	0 (0%)	0 (0%)	0 (0%)	1 (.3%)	0 (0%)	0 (0%)	0 (0%)	0 (0%)	0 (0%)	0 (0%)	1 (.6%)	0 (0%)
child welfare (incl. Immunization)	188 (48.2%)	69 (35.6%)	88 (43.8%)	198 (66.2%)	121 (38.4%)	130 (52.8%)	126 (60.3%)	53 (53%)	36 (47.4%)	61 (57.5%)	90 (52%)	58 (55.8%)
Other	0 (0%)	1 (.5%)	2 (1%)	11 (3.7%)	10 (3.2%)	2 (.8%)	0 (0%)	2 (2%)	0 (0%)	0 (0%)	5 (2.9%)	0 (0%)
Any MCH service	232 (59.5%)	133 (68.6%)	118 (58.7%)	71 (23.7%)	112 (35.6%)	116 (47.2%)	68 (32.5%)	47 (47%)	42 (55.3%)	52 (49.1%)	75 (43.4%)	55 (52.9%)
**Integrated HIV/STI & MCH services received**												
neither	156 (40%)	61 (31.4%)	83 (41.3%)	228 (76.3%)	190 (60.3%)	122 (49.6%)	140 (67%)	51 (51%)	34 (44.7%)	53 (50%)	88 (50.9%)	49 (47.1%)
MCH only	229 (58.7%)	113 (58.2%)	102 (50.7%)	68 (22.7%)	92 (29.2%)	112 (45.5%)	68 (32.5%)	40 (40%)	28 (36.8%)	36 (34%)	30 (17.3%)	53 (51%)
HIV/STI only	2 (.5%)	0 (0%)	0 (0%)	0 (0%)	13 (4.1%)	8 (3.3%)	1 (.5%)	2 (2%)	0 (0%)	1 (.9%)	10 (5.8%)	0 (0%)
HIV/STI & MCH	3 (.8%)	20 (10.3%)	16 (8%)	3 (1%)	20 (6.3%)	4 (1.6%)	0 (0%)	7 (7%)	14 (18.4%)	16 (15.1%)	45 (26%)	2 (1.9%)
**Receipt of any HIV/STI service plus...**												
Family Planning Provision	1 (.3%)	10 (5.2%)	0 (0%)	0 (0%)	7 (2.2%)	1 (.4%)	0 (0%)	5 (5%)	4 (5.3%)	7 (6.6%)	32 (18.5%)	1 (1%)
Family Planning Counselling	1 (.3%)	10 (5.2%)	5 (2.5%)	1 (.3%)	11 (3.5%)	1 (.4%)	0 (0%)	5 (5%)	2 (2.6%)	2 (1.9%)	30 (17.3%)	0 (0%)
ANC	2 (.5%)	4 (2.1%)	11 (5.5%)	2 (.7%)	8 (2.5%)	3 (1.2%)	0 (0%)	2 (2%)	10 (13.2%)	9 (8.5%)	7 (4%)	1 (1%)
PNC (mother or baby)	0 (0%)	0 (0%)	3 (1.5%)	1 (.3%)	4 (1.3%)	0 (0%)	0 (0%)	1 (1%)	0 (0%)	0 (0%)	5 (2.9%)	0 (0%)
cervical screening	0 (0%)	0 (0%)	0 (0%)	0 (0%)	0 (0%)	0 (0%)	0 (0%)	0 (0%)	0 (0%)	0 (0%)	1 (.6%)	0 (0%)
child welfare (incl. Immunization)	1 (.3%)	1 (.5%)	3 (1.5%)	1 (.3%)	5 (1.6%)	0 (0%)	0 (0%)	2 (2%)	0 (0%)	2 (1.9%)	6 (3.5%)	0 (0%)
Other RH/MCH service	0 (0%)	0 (0%)	1 (.5%)	0 (0%)	1 (.3%)	0 (0%)	0 (0%)	0 (0%)	0 (0%)	0 (0%)	3 (1.7%)	0 (0%)

	Facility C						Facility D					
	Jun2009	Jan2010	Jun2010	Jan2011	Jun2011	Jan2012	Jun2009	Jan2010	Jun2010	Jan2011	Jun2011	Jan2012
**Visits tracked**	89	81	76	56	64	72	135	135	103	135	109	76
**Client age (years)**												
Mean [sd]	29.4 [9]	29.2 [7.6]	27.2 [7.6]	24.1 [5.6]	26.1 [6.8]	27.6 [6.8]	27 [6.9]	32 [10.2]	26 [5.7]	27.3 [6.2]	26.4 [6.6]	25.8 [6.2]
Missing	0 (0%)	0 (0%)	0 (0%)	1 (1.8%)	31 (48.4%)	1 (1.4%)	0 (0%)	0 (0%)	0 (0%)	0 (0%)	0 (0%)	0 (0%)
**Services received per client**												
none	0 (0%)	0 (0%)	0 (0%)	0 (0%)	0 (0%)	0 (0%)	0 (0%)	3 (2.2%)	0 (0%)	1 (.7%)	2 (1.8%)	3 (3.9%)
one	46 (51.7%)	44 (54.3%)	46 (60.5%)	36 (64.3%)	4 (6.3%)	36 (50%)	37 (27.4%)	115 (85.2%)	62 (60.2%)	62 (45.9%)	36 (33%)	44 (57.9%)
two or more	43 (48.3%)	37 (45.7%)	30 (39.5%)	20 (35.7%)	60 (93.8%)	36 (50%)	98 (72.6%)	17 (12.6%)	41 (39.8%)	72 (53.3%)	71 (65.1%)	29 (38.2%)
**Visits where at least one services was received...**												
Mean [sd] services received	1.7 [.9]	2.5 [1.9]	1.6 [.9]	1.8 [1.3]	2.8 [1.2]	2 [1.3]	1.8 [.6]	1.2 [.4]	1.7 [1.1]	1.7 [1]	2.1 [1.2]	1.5 [.9]
Mean [sd] providers seen	1.3 [.6]	1.8 [1]	1.1 [.3]	1.2 [.4]	1.5 [.9]	1.7 [.9]	1 [0]	1 [0]	1.2 [.5]	1.3 [.7]	1.4 [.8]	1.2 [.7]
**Top 3 services provided**												
1	Child welfare	FP provision	Child welfare	Child welfare	Child welfare	Child welfare	Child welfare	Child welfare	Child welfare	Child welfare	Child welfare	Child welfare
2	HIV treatment	HIV testing	FP provision	ANC	Other services	ANC	ANC	HIV treatment	ANC	ANC	ANC	ANC
3	ANC	FP counselling	ANC	general counsel	lab	lab	FP provision	ANC	FP provision	FP provision	FP counselling	Other services
**HIV/STI services received**												
HIV testing	2 (2.2%)	29 (35.8%)	3 (3.9%)	6 (10.7%)	0 (0%)	1 (1.4%)	10 (7.4%)	0 (0%)	6 (5.8%)	3 (2.2%)	8 (7.3%)	2 (2.6%)
HIV counselling	8 (9%)	25 (30.9%)	3 (3.9%)	6 (10.7%)	0 (0%)	1 (1.4%)	8 (5.9%)	1 (.7%)	4 (3.9%)	0 (0%)	0 (0%)	0 (0%)
HIV treatment	25 (28.1%)	13 (16%)	8 (10.5%)	0 (0%)	3 (4.7%)	1 (1.4%)	3 (2.2%)	51 (37.8%)	0 (0%)	0 (0%)	2 (1.8%)	0 (0%)
HIV PMTCT	0 (0%)	1 (1.2%)	2 (2.6%)	6 (10.7%)	7 (10.9%)	3 (4.2%)	0 (0%)	0 (0%)	0 (0%)	4 (3%)	3 (2.8%)	0 (0%)
Other HIV/STI service	0 (0%)	0 (0%)	0 (0%)	0 (0%)	0 (0%)	0 (0%)	0 (0%)	0 (0%)	0 (0%)	0 (0%)	0 (0%)	0 (0%)
STI counselling or testing	0 (0%)	0 (0%)	0 (0%)	0 (0%)	0 (0%)	1 (1.4%)	0 (0%)	0 (0%)	0 (0%)	0 (0%)	1 (.9%)	0 (0%)
Any HIV/STI services	32 (36%)	42 (51.9%)	11 (14.5%)	6 (10.7%)	3 (4.7%)	3 (4.2%)	11 (8.1%)	51 (37.8%)	6 (5.8%)	3 (2.2%)	11 (10.1%)	2 (2.6%)
**MCH services received**												
Family Planning Provision	20 (22.5%)	38 (46.9%)	25 (32.9%)	0 (0%)	14 (21.9%)	5 (6.9%)	17 (12.6%)	7 (5.2%)	22 (21.4%)	23 (17%)	13 (11.9%)	4 (5.3%)
Family Planning Counselling	11 (12.4%)	26 (32.1%)	16 (21.1%)	2 (3.6%)	14 (21.9%)	6 (8.3%)	14 (10.4%)	2 (1.5%)	6 (5.8%)	1 (.7%)	15 (13.8%)	0 (0%)
ANC	18 (20.2%)	14 (17.3%)	17 (22.4%)	23 (41.1%)	15 (23.4%)	21 (29.2%)	26 (19.3%)	8 (5.9%)	19 (18.4%)	32 (23.7%)	35 (32.1%)	12 (15.8%)
PNC (mother or baby)	3 (3.4%)	3 (3.7%)	1 (1.3%)	0 (0%)	6 (9.4%)	1 (1.4%)	0 (0%)	1 (.7%)	9 (8.7%)	4 (3%)	8 (7.3%)	1 (1.3%)
cervical screening	0 (0%)	0 (0%)	0 (0%)	0 (0%)	0 (0%)	0 (0%)	0 (0%)	0 (0%)	0 (0%)	0 (0%)	0 (0%)	0 (0%)
child welfare (incl. Immunization)	35 (39.3%)	15 (18.5%)	33 (43.4%)	33 (58.9%)	35 (54.7%)	44 (61.1%)	88 (65.2%)	66 (48.9%)	65 (63.1%)	78 (57.8%)	61 (56%)	55 (72.4%)
Other	0 (0%)	0 (0%)	0 (0%)	0 (0%)	0 (0%)	0 (0%)	0 (0%)	0 (0%)	0 (0%)	0 (0%)	0 (0%)	0 (0%)
Any MCH service	47 (52.8%)	55 (67.9%)	42 (55.3%)	24 (42.9%)	35 (54.7%)	31 (43.1%)	43 (31.9%)	15 (11.1%)	51 (49.5%)	60 (44.4%)	55 (50.5%)	17 (22.4%)
**Integrated HIV/STI & MCH services received**												
neither	25 (28.1%)	14 (17.3%)	26 (34.2%)	32 (57.1%)	28 (43.8%)	40 (55.6%)	92 (68.1%)	69 (51.1%)	52 (50.5%)	75 (55.6%)	53 (48.6%)	59 (77.6%)
MCH only	32 (36%)	25 (30.9%)	39 (51.3%)	18 (32.1%)	33 (51.6%)	29 (40.3%)	32 (23.7%)	15 (11.1%)	45 (43.7%)	57 (42.2%)	45 (41.3%)	15 (19.7%)
HIV/STI only	17 (19.1%)	12 (14.8%)	8 (10.5%)	0 (0%)	1 (1.6%)	1 (1.4%)	0 (0%)	51 (37.8%)	0 (0%)	0 (0%)	1 (.9%)	0 (0%)
HIV/STI & MCH	15 (16.9%)	30 (37%)	3 (3.9%)	6 (10.7%)	2 (3.1%)	2 (2.8%)	11 (8.1%)	0 (0%)	6 (5.8%)	3 (2.2%)	10 (9.2%)	2 (2.6%)
**Receipt of any HIV/STI service plus...**												
Family Planning Provision	12 (13.5%)	30 (37%)	2 (2.6%)	0 (0%)	1 (1.6%)	2 (2.8%)	0 (0%)	0 (0%)	4 (3.9%)	0 (0%)	3 (2.8%)	1 (1.3%)
Family Planning Counselling	7 (7.9%)	23 (28.4%)	1 (1.3%)	0 (0%)	2 (3.1%)	2 (2.8%)	0 (0%)	0 (0%)	2 (1.9%)	0 (0%)	5 (4.6%)	0 (0%)
ANC	1 (1.1%)	0 (0%)	1 (1.3%)	6 (10.7%)	0 (0%)	0 (0%)	11 (8.1%)	0 (0%)	2 (1.9%)	3 (2.2%)	7 (6.4%)	1 (1.3%)
PNC (mother or baby)	0 (0%)	0 (0%)	0 (0%)	0 (0%)	0 (0%)	0 (0%)	0 (0%)	0 (0%)	0 (0%)	0 (0%)	1 (.9%)	0 (0%)
cervical screening	0 (0%)	0 (0%)	0 (0%)	0 (0%)	0 (0%)	0 (0%)	0 (0%)	0 (0%)	0 (0%)	0 (0%)	0 (0%)	0 (0%)
child welfare (incl. Immunization)	5 (5.6%)	2 (2.5%)	0 (0%)	0 (0%)	1 (1.6%)	1 (1.4%)	0 (0%)	0 (0%)	0 (0%)	0 (0%)	3 (2.8%)	0 (0%)
Other RH/MCH service	0 (0%)	0 (0%)	0 (0%)	0 (0%)	0 (0%)	0 (0%)	0 (0%)	0 (0%)	0 (0%)	0 (0%)	0 (0%)	0 (0%)

	Facility E						Facility F					
	Jun2009	Jan2010	Jun2010	Jan2011	Jun2011	Jan2012	Jun2009	Jan2010	Jun2010	Jan2011	Jun2011	Jan2012
**Visits tracked**	106	134	131	155	100	134	100	117	86	140	69	101
**Client age (years)**												
Mean [sd]	25.7 [6.6]	26.4 [7.3]	26.1 [6]	27 [6.8]	26.1 [6.2]	26 [6.2]	26.2 [6.8]	28.1 [7]	27.7 [7]	27 [6.4]	27.3 [7.2]	26.5 [7.5]
Missing	0 (0%)	0 (0%)	1 (.8%)	0 (0%)	9 (9%)	0 (0%)	1 (1%)	1 (.9%)	0 (0%)	0 (0%)	1 (1.4%)	0 (0%)
**Services received per client**												
none	0 (0%)	1 (.7%)	0 (0%)	0 (0%)	0 (0%)	0 (0%)	0 (0%)	1 (.9%)	0 (0%)	0 (0%)	0 (0%)	0 (0%)
one	30 (28.3%)	32 (23.9%)	32 (24.4%)	45 (29%)	33 (33%)	95 (70.9%)	56 (56%)	31 (26.5%)	49 (57%)	51 (36.4%)	21 (30.4%)	70 (69.3%)
two or more	76 (71.7%)	101 (75.4%)	99 (75.6%)	110 (71%)	67 (67%)	39 (29.1%)	44 (44%)	85 (72.6%)	37 (43%)	89 (63.6%)	48 (69.6%)	31 (30.7%)
**Visits where at least one services was received...**												
Mean [sd] services received	1.8 [.6]	2.8 [1.8]	2.5 [1.6]	2 [.8]	2 [1]	1.3 [.5]	1.8 [1.2]	3.4 [2.2]	2 [1.5]	2.6 [1.7]	2.5 [1.5]	1.5 [.9]
Mean [sd] providers seen	1 [0]	1.2 [.6]	1.1 [.5]	1.2 [.5]	1 [.2]	1 [.2]	1.4 [.8]	1.4 [.8]	1.4 [.8]	1.7 [1]	1.9 [.9]	1.1 [.4]
**Top 3 services provided**												
1	Child welfare	Child welfare	Child welfare	Child welfare	Child welfare	Child welfare	Child welfare	Child welfare	Child welfare	Child welfare	Child welfare	Child welfare
2	ANC	general counsel	ANC	ANC	ANC	ANC	ANC	general counsel	ANC	ANC	ANC	ANC
3	FP provision	ANC	general counsel	Other services	general counsel	FP provision	FP provision	PNC	FP provision	lab	FP counselling	PNC
**HIV/STI services received**												
HIV testing	8 (7.5%)	4 (3%)	19 (14.5%)	2 (1.3%)	10 (10%)	1 (.7%)	7 (7%)	25 (21.4%)	15 (17.4%)	22 (15.7%)	9 (13%)	1 (1%)
HIV counselling	8 (7.5%)	14 (10.4%)	21 (16%)	3 (1.9%)	13 (13%)	0 (0%)	5 (5%)	25 (21.4%)	15 (17.4%)	22 (15.7%)	9 (13%)	1 (1%)
HIV treatment	1 (.9%)	1 (.7%)	2 (1.5%)	0 (0%)	1 (1%)	0 (0%)	0 (0%)	2 (1.7%)	0 (0%)	0 (0%)	1 (1.4%)	1 (1%)
HIV PMTCT	0 (0%)	2 (1.5%)	16 (12.2%)	12 (7.7%)	8 (8%)	0 (0%)	0 (0%)	8 (6.8%)	0 (0%)	0 (0%)	0 (0%)	0 (0%)
Other HIV/STI service	0 (0%)	0 (0%)	0 (0%)	0 (0%)	0 (0%)	0 (0%)	0 (0%)	0 (0%)	0 (0%)	0 (0%)	0 (0%)	0 (0%)
STI counselling or testing	0 (0%)	0 (0%)	0 (0%)	0 (0%)	1 (1%)	0 (0%)	0 (0%)	2 (1.7%)	0 (0%)	0 (0%)	0 (0%)	0 (0%)
Any HIV/STI services	9 (8.5%)	14 (10.4%)	21 (16%)	3 (1.9%)	15 (15%)	1 (.7%)	7 (7%)	27 (23.1%)	15 (17.4%)	22 (15.7%)	10 (14.5%)	1 (1%)
**MCH services received**												
Family Planning Provision	10 (9.4%)	18 (13.4%)	12 (9.2%)	11 (7.1%)	13 (13%)	7 (5.2%)	11 (11%)	21 (17.9%)	15 (17.4%)	11 (7.9%)	8 (11.6%)	6 (5.9%)
Family Planning Counselling	6 (5.7%)	30 (22.4%)	19 (14.5%)	9 (5.8%)	11 (11%)	4 (3%)	4 (4%)	43 (36.8%)	6 (7%)	21 (15%)	13 (18.8%)	2 (2%)
ANC	31 (29.2%)	36 (26.9%)	43 (32.8%)	43 (27.7%)	26 (26%)	39 (29.1%)	17 (17%)	15 (12.8%)	20 (23.3%)	27 (19.3%)	16 (23.2%)	18 (17.8%)
PNC (mother or baby)	2 (1.9%)	15 (11.2%)	4 (3.1%)	1 (.6%)	3 (3%)	0 (0%)	0 (0%)	22 (18.8%)	0 (0%)	16 (11.4%)	7 (10.1%)	7 (6.9%)
cervical screening	0 (0%)	0 (0%)	0 (0%)	0 (0%)	0 (0%)	0 (0%)	0 (0%)	0 (0%)	0 (0%)	0 (0%)	0 (0%)	0 (0%)
child welfare (incl. Immunization)	65 (61.3%)	82 (61.2%)	73 (55.7%)	99 (63.9%)	48 (48%)	87 (64.9%)	73 (73%)	87 (74.4%)	53 (61.6%)	107 (76.4%)	44 (63.8%)	77 (76.2%)
Other	0 (0%)	0 (0%)	0 (0%)	0 (0%)	0 (0%)	0 (0%)	0 (0%)	0 (0%)	0 (0%)	0 (0%)	0 (0%)	0 (0%)
Any MCH service	44 (41.5%)	73 (54.5%)	61 (46.6%)	55 (35.5%)	44 (44%)	47 (35.1%)	28 (28%)	70 (59.8%)	36 (41.9%)	54 (38.6%)	34 (49.3%)	31 (30.7%)
**Integrated HIV/STI & MCH services received**												
neither	62 (58.5%)	61 (45.5%)	69 (52.7%)	99 (63.9%)	48 (48%)	87 (64.9%)	72 (72%)	46 (39.3%)	50 (58.1%)	86 (61.4%)	34 (49.3%)	70 (69.3%)
MCH only	35 (33%)	59 (44%)	41 (31.3%)	53 (34.2%)	37 (37%)	46 (34.3%)	21 (21%)	44 (37.6%)	21 (24.4%)	32 (22.9%)	25 (36.2%)	30 (29.7%)
HIV/STI only	0 (0%)	0 (0%)	1 (.8%)	1 (.6%)	8 (8%)	0 (0%)	0 (0%)	1 (.9%)	0 (0%)	0 (0%)	1 (1.4%)	0 (0%)
HIV/STI & MCH	9 (8.5%)	14 (10.4%)	20 (15.3%)	2 (1.3%)	7 (7%)	1 (.7%)	7 (7%)	26 (22.2%)	15 (17.4%)	22 (15.7%)	9 (13%)	1 (1%)
**Receipt of any HIV/STI service plus...**												
Family Planning Provision	0 (0%)	3 (2.2%)	1 (.8%)	0 (0%)	3 (3%)	0 (0%)	2 (2%)	15 (12.8%)	6 (7%)	9 (6.4%)	3 (4.3%)	0 (0%)
Family Planning Counselling	0 (0%)	10 (7.5%)	11 (8.4%)	0 (0%)	2 (2%)	0 (0%)	1 (1%)	12 (10.3%)	3 (3.5%)	7 (5%)	2 (2.9%)	0 (0%)
ANC	9 (8.5%)	8 (6%)	15 (11.5%)	2 (1.3%)	1 (1%)	1 (.7%)	5 (5%)	8 (6.8%)	9 (10.5%)	13 (9.3%)	7 (10.1%)	0 (0%)
PNC (mother or baby)	0 (0%)	2 (1.5%)	3 (2.3%)	0 (0%)	3 (3%)	0 (0%)	0 (0%)	2 (1.7%)	0 (0%)	0 (0%)	0 (0%)	1 (1%)
cervical screening	0 (0%)	0 (0%)	0 (0%)	0 (0%)	0 (0%)	0 (0%)	0 (0%)	0 (0%)	0 (0%)	0 (0%)	0 (0%)	0 (0%)
child welfare (incl. Immunization)	0 (0%)	4 (3%)	4 (3.1%)	1 (.6%)	4 (4%)	0 (0%)	0 (0%)	10 (8.5%)	0 (0%)	3 (2.1%)	1 (1.4%)	1 (1%)
Other RH/MCH service	0 (0%)	0 (0%)	0 (0%)	0 (0%)	0 (0%)	0 (0%)	0 (0%)	0 (0%)	0 (0%)	0 (0%)	0 (0%)	0 (0%)

District E2 Facilities (Facilities G–L)

	Facility G						Facility H					
	Jun2009	Jan2010	Jun2010	Jan2011	Jun2011	Jan2012	Jun2009	Jan2010	Jun2010	Jan2011	Jun2011	Jan2012
**Visits tracked**	202	186	235	386	301	316	235	182	106	194	130	131
**Client age (years)**												
Mean [sd]	27.3 [6.6]	26.8 [6.2]	27.9 [6.4]	26.6 [5.8]	26.8 [5.7]	27.7 [8]	26.7 [6.3]	28.7 [7.8]	27.1 [6.7]	27 [6.4]	26.3 [5.9]	27.6 [6.5]
Missing	0 (0%)	1 (.5%)	3 (1.3%)	0 (0%)	3 (1%)	2 (.6%)	0 (0%)	6 (3.3%)	0 (0%)	0 (0%)	1 (.8%)	0 (0%)
**Services received per client**												
none	0 (0%)	0 (0%)	0 (0%)	1 (.3%)	0 (0%)	0 (0%)	0 (0%)	0 (0%)	0 (0%)	0 (0%)	1 (.8%)	0 (0%)
one	105 (52%)	81 (43.5%)	115 (48.9%)	227 (58.8%)	186 (61.8%)	65 (20.6%)	26 (11.1%)	12 (6.6%)	18 (17%)	17 (8.8%)	37 (28.5%)	28 (21.4%)
two or more	97 (48%)	105 (56.5%)	120 (51.1%)	158 (40.9%)	115 (38.2%)	251 (79.4%)	209 (88.9%)	170 (93.4%)	88 (83%)	177 (91.2%)	92 (70.8%)	103 (78.6%)
**Visits where at least one services was received...**												
Mean [sd] services received	1.9 [1.2]	2.2 [1.4]	1.9 [1.1]	1.6 [.9]	1.4 [.6]	2.8 [1.6]	2.9 [1.1]	3.6 [1.4]	3.7 [1.6]	4.7 [2.1]	3.7 [2.2]	3.9 [2.3]
Mean [sd] providers seen	1.4 [.7]	1.2 [.6]	1.4 [.7]	1.3 [.6]	1.4 [.5]	2 [.8]	1 [.2]	1.5 [.6]	1.3 [.5]	1.1 [.4]	1.1 [.5]	1.5 [.6]
**Top 3 services provided**												
1	Child welfare	Child welfare	Child welfare	Child welfare	Child welfare	Other services	Child welfare	Child welfare	Child welfare	Child welfare	Child welfare	Child welfare
2	ANC	FP counselling	ANC	Other services	FP provision	Child welfare	HIV counselling	pharmacy	HIV counselling	HIV counselling	general counsel	general counsel
3	FP provision	FP provision	FP provision	pharmacy	pharmacy	ANC	FP counselling	Other services	FP counselling	STI	HIV counselling	FP counselling
**HIV/STI services received**												
HIV testing	19 (9.4%)	33 (17.7%)	22 (9.4%)	4 (1%)	2 (.7%)	6 (1.9%)	18 (7.7%)	19 (10.4%)	11 (10.4%)	14 (7.2%)	9 (6.9%)	1 (.8%)
HIV counselling	28 (13.9%)	40 (21.5%)	43 (18.3%)	9 (2.3%)	0 (0%)	10 (3.2%)	132 (56.2%)	46 (25.3%)	65 (61.3%)	143 (73.7%)	65 (50%)	58 (44.3%)
HIV treatment	10 (5%)	2 (1.1%)	8 (3.4%)	3 (.8%)	2 (.7%)	9 (2.8%)	3 (1.3%)	1 (.5%)	1 (.9%)	0 (0%)	3 (2.3%)	8 (6.1%)
HIV PMTCT	0 (0%)	3 (1.6%)	11 (4.7%)	0 (0%)	7 (2.3%)	15 (4.7%)	0 (0%)	2 (1.1%)	0 (0%)	12 (6.2%)	0 (0%)	3 (2.3%)
Other HIV/STI service	0 (0%)	0 (0%)	0 (0%)	0 (0%)	0 (0%)	0 (0%)	0 (0%)	0 (0%)	0 (0%)	0 (0%)	0 (0%)	0 (0%)
STI counselling or testing	1 (.5%)	0 (0%)	0 (0%)	0 (0%)	0 (0%)	0 (0%)	0 (0%)	1 (.5%)	10 (9.4%)	128 (66%)	4 (3.1%)	53 (40.5%)
Any HIV/STI services	34 (16.8%)	42 (22.6%)	47 (20%)	12 (3.1%)	4 (1.3%)	18 (5.7%)	147 (62.6%)	57 (31.3%)	74 (69.8%)	148 (76.3%)	69 (53.1%)	66 (50.4%)
**MCH services received**												
Family Planning Provision	48 (23.8%)	60 (32.3%)	64 (27.2%)	55 (14.2%)	42 (14%)	39 (12.3%)	73 (31.1%)	47 (25.8%)	37 (34.9%)	55 (28.4%)	9 (6.9%)	17 (13%)
Family Planning Counselling	47 (23.3%)	60 (32.3%)	30 (12.8%)	16 (4.1%)	12 (4%)	35 (11.1%)	110 (46.8%)	85 (46.7%)	55 (51.9%)	95 (49%)	57 (43.8%)	63 (48.1%)
ANC	45 (22.3%)	30 (16.1%)	64 (27.2%)	49 (12.7%)	24 (8%)	58 (18.4%)	33 (14%)	12 (6.6%)	21 (19.8%)	22 (11.3%)	17 (13.1%)	16 (12.2%)
PNC (mother or baby)	14 (6.9%)	1 (.5%)	0 (0%)	2 (.5%)	0 (0%)	2 (.6%)	14 (6%)	4 (2.2%)	8 (7.5%)	16 (8.2%)	6 (4.6%)	2 (1.5%)
cervical screening	0 (0%)	5 (2.7%)	1 (.4%)	1 (.3%)	1 (.3%)	0 (0%)	0 (0%)	0 (0%)	0 (0%)	1 (.5%)	0 (0%)	0 (0%)
child welfare (incl. Immunization)	96 (47.5%)	98 (52.7%)	99 (42.1%)	171 (44.3%)	218 (72.4%)	150 (47.5%)	173 (73.6%)	166 (91.2%)	80 (75.5%)	144 (74.2%)	103 (79.2%)	105 (80.2%)
Other	0 (0%)	0 (0%)	0 (0%)	0 (0%)	1 (.3%)	2 (.6%)	0 (0%)	0 (0%)	0 (0%)	0 (0%)	0 (0%)	0 (0%)
Any MCH service	129 (63.9%)	95 (51.1%)	132 (56.2%)	117 (30.3%)	76 (25.2%)	117 (37%)	189 (80.4%)	112 (61.5%)	95 (89.6%)	151 (77.8%)	72 (55.4%)	83 (63.4%)
**Integrated HIV/STI & MCH services received**												
neither	65 (32.2%)	89 (47.8%)	96 (40.9%)	269 (69.7%)	222 (73.8%)	192 (60.8%)	39 (16.6%)	58 (31.9%)	8 (7.5%)	32 (16.5%)	48 (36.9%)	44 (33.6%)
MCH only	103 (51%)	55 (29.6%)	92 (39.1%)	105 (27.2%)	75 (24.9%)	106 (33.5%)	49 (20.9%)	67 (36.8%)	24 (22.6%)	14 (7.2%)	13 (10%)	21 (16%)
HIV/STI only	8 (4%)	2 (1.1%)	7 (3%)	0 (0%)	3 (1%)	7 (2.2%)	7 (3%)	12 (6.6%)	3 (2.8%)	11 (5.7%)	10 (7.7%)	4 (3.1%)
HIV/STI & MCH	26 (12.9%)	40 (21.5%)	40 (17%)	12 (3.1%)	1 (.3%)	11 (3.5%)	140 (59.6%)	45 (24.7%)	71 (67%)	137 (70.6%)	59 (45.4%)	62 (47.3%)
**Receipt of any HIV/STI service plus...**												
Family Planning Provision	4 (2%)	38 (20.4%)	31 (13.2%)	0 (0%)	0 (0%)	1 (.3%)	54 (23%)	14 (7.7%)	32 (30.2%)	49 (25.3%)	8 (6.2%)	9 (6.9%)
Family Planning Counselling	10 (5%)	38 (20.4%)	14 (6%)	0 (0%)	1 (.3%)	2 (.6%)	87 (37%)	33 (18.1%)	48 (45.3%)	90 (46.4%)	53 (40.8%)	58 (44.3%)
ANC	15 (7.4%)	2 (1.1%)	9 (3.8%)	12 (3.1%)	0 (0%)	8 (2.5%)	17 (7.2%)	4 (2.2%)	6 (5.7%)	20 (10.3%)	7 (5.4%)	5 (3.8%)
PNC (mother or baby)	2 (1%)	1 (.5%)	0 (0%)	0 (0%)	0 (0%)	1 (.3%)	3 (1.3%)	1 (.5%)	7 (6.6%)	10 (5.2%)	6 (4.6%)	2 (1.5%)
cervical screening	0 (0%)	2 (1.1%)	0 (0%)	0 (0%)	0 (0%)	0 (0%)	0 (0%)	0 (0%)	0 (0%)	1 (.5%)	0 (0%)	0 (0%)
child welfare (incl. Immunization)	7 (3.5%)	5 (2.7%)	9 (3.8%)	0 (0%)	3 (1%)	4 (1.3%)	114 (48.5%)	53 (29.1%)	64 (60.4%)	115 (59.3%)	57 (43.8%)	59 (45%)
Other RH/MCH service	0 (0%)	0 (0%)	0 (0%)	0 (0%)	0 (0%)	0 (0%)	0 (0%)	0 (0%)	0 (0%)	0 (0%)	0 (0%)	0 (0%)

	Facility I						Facility J					
	Jun2009	Jan2010	Jun2010	Jan2011	Jun2011	Jan2012	Jun2009	Jan2010	Jun2010	Jan2011	Jun2011	Jan2012
**Visits tracked**	115	153	112	125	87	97	87	125	109	94	70	79
**Client age (years)**												
Mean [sd]	27.5 [7.3]	26.2 [8.3]	25.4 [5.5]	26.7 [7.1]	24.7 [5.4]	26.6 [6.6]	27.5 [6.7]	26.7 [8.2]	27.6 [7.6]	26.7 [6.6]	27.7 [10.5]	26.7 [4.9]
Missing	0 (0%)	0 (0%)	0 (0%)	0 (0%)	0 (0%)	1 (1%)	0 (0%)	0 (0%)	0 (0%)	0 (0%)	49 (70%)	2 (2.5%)
**Services received per client**												
none	0 (0%)	0 (0%)	0 (0%)	0 (0%)	0 (0%)	0 (0%)	0 (0%)	0 (0%)	0 (0%)	0 (0%)	0 (0%)	0 (0%)
one	61 (53%)	11 (7.2%)	48 (42.9%)	64 (51.2%)	22 (25.3%)	65 (67%)	29 (33.3%)	5 (4%)	18 (16.5%)	41 (43.6%)	17 (24.3%)	54 (68.4%)
two or more	54 (47%)	142 (92.8%)	64 (57.1%)	61 (48.8%)	65 (74.7%)	32 (33%)	58 (66.7%)	120 (96%)	91 (83.5%)	53 (56.4%)	53 (75.7%)	25 (31.6%)
**Visits where at least one services was received...**												
Mean [sd] services received	1.6 [.8]	2.9 [1.1]	2.1 [1.2]	2.1 [1.2]	2.8 [1.4]	1.4 [.5]	2.3 [1.3]	3.4 [1.5]	2.7 [1.3]	2.4 [1.4]	2.2 [.9]	1.6 [1.2]
Mean [sd] providers seen	1.5 [.6]	2.4 [.8]	1.7 [1]	1.8 [1]	1.2 [.6]	1 [.2]	1 [.1]	2.7 [1.1]	1.6 [.9]	1.8 [.9]	1.7 [.9]	1.1 [.4]
**Top 3 services provided**												
1	Child welfare	Child welfare	Child welfare	Child welfare	Child welfare	Child welfare	Child welfare	general counsel	Child welfare	Child welfare	Child welfare	Child welfare
2	FP counselling	pharmacy	pharmacy	Other services	general counsel	ANC	FP counselling	Child welfare	Other services	Other services	Other services	ANC
3	ANC	Other services	Other services	pharmacy	Other services	FP counselling	FP provision	pharmacy	pharmacy	pharmacy	pharmacy	FP counselling
**HIV/STI services received**												
HIV testing	0 (0%)	1 (.7%)	2 (1.8%)	8 (6.4%)	0 (0%)	0 (0%)	10 (11.5%)	4 (3.2%)	5 (4.6%)	4 (4.3%)	1 (1.4%)	2 (2.5%)
HIV counselling	0 (0%)	6 (3.9%)	2 (1.8%)	9 (7.2%)	0 (0%)	0 (0%)	12 (13.8%)	13 (10.4%)	6 (5.5%)	4 (4.3%)	0 (0%)	3 (3.8%)
HIV treatment	0 (0%)	0 (0%)	0 (0%)	2 (1.6%)	0 (0%)	0 (0%)	0 (0%)	3 (2.4%)	1 (.9%)	1 (1.1%)	9 (12.9%)	1 (1.3%)
HIV PMTCT	0 (0%)	6 (3.9%)	6 (5.4%)	4 (3.2%)	1 (1.1%)	0 (0%)	0 (0%)	5 (4%)	8 (7.3%)	2 (2.1%)	0 (0%)	4 (5.1%)
Other HIV/STI service	0 (0%)	0 (0%)	0 (0%)	0 (0%)	0 (0%)	0 (0%)	0 (0%)	0 (0%)	0 (0%)	0 (0%)	0 (0%)	0 (0%)
STI counselling or testing	0 (0%)	1 (.7%)	1 (.9%)	0 (0%)	0 (0%)	0 (0%)	9 (10.3%)	0 (0%)	1 (.9%)	0 (0%)	0 (0%)	0 (0%)
Any HIV/STI services	0 (0%)	8 (5.2%)	4 (3.6%)	11 (8.8%)	0 (0%)	0 (0%)	16 (18.4%)	15 (12%)	11 (10.1%)	5 (5.3%)	10 (14.3%)	4 (5.1%)
**MCH services received**												
Family Planning Provision	1 (.9%)	10 (6.5%)	6 (5.4%)	11 (8.8%)	11 (12.6%)	5 (5.2%)	32 (36.8%)	17 (13.6%)	13 (11.9%)	20 (21.3%)	8 (11.4%)	6 (7.6%)
Family Planning Counselling	10 (8.7%)	18 (11.8%)	5 (4.5%)	6 (4.8%)	27 (31%)	5 (5.2%)	40 (46%)	13 (10.4%)	22 (20.2%)	8 (8.5%)	7 (10%)	12 (15.2%)
ANC	6 (5.2%)	17 (11.1%)	17 (15.2%)	22 (17.6%)	6 (6.9%)	25 (25.8%)	21 (24.1%)	9 (7.2%)	21 (19.3%)	11 (11.7%)	3 (4.3%)	14 (17.7%)
PNC (mother or baby)	0 (0%)	0 (0%)	7 (6.3%)	1 (.8%)	0 (0%)	0 (0%)	12 (13.8%)	2 (1.6%)	1 (.9%)	0 (0%)	0 (0%)	1 (1.3%)
cervical screening	0 (0%)	0 (0%)	0 (0%)	0 (0%)	0 (0%)	0 (0%)	0 (0%)	0 (0%)	1 (.9%)	0 (0%)	0 (0%)	0 (0%)
child welfare (incl. Immunization)	111 (96.5%)	106 (69.3%)	83 (74.1%)	94 (75.2%)	74 (85.1%)	67 (69.1%)	39 (44.8%)	79 (63.2%)	69 (63.3%)	62 (66%)	53 (75.7%)	51 (64.6%)
Other	0 (0%)	0 (0%)	0 (0%)	0 (0%)	0 (0%)	0 (0%)	0 (0%)	0 (0%)	0 (0%)	0 (0%)	0 (0%)	0 (0%)
Any MCH service	17 (14.8%)	36 (23.5%)	30 (26.8%)	34 (27.2%)	35 (40.2%)	30 (30.9%)	73 (83.9%)	34 (27.2%)	44 (40.4%)	34 (36.2%)	16 (22.9%)	28 (35.4%)
**Integrated HIV/STI & MCH services received**												
neither	98 (85.2%)	116 (75.8%)	81 (72.3%)	91 (72.8%)	52 (59.8%)	67 (69.1%)	14 (16.1%)	86 (68.8%)	62 (56.9%)	59 (62.8%)	50 (71.4%)	51 (64.6%)
MCH only	17 (14.8%)	29 (19%)	27 (24.1%)	23 (18.4%)	35 (40.2%)	30 (30.9%)	57 (65.5%)	24 (19.2%)	36 (33%)	30 (31.9%)	10 (14.3%)	24 (30.4%)
HIV/STI only	0 (0%)	1 (.7%)	1 (.9%)	0 (0%)	0 (0%)	0 (0%)	0 (0%)	5 (4%)	3 (2.8%)	1 (1.1%)	4 (5.7%)	0 (0%)
HIV/STI & MCH	0 (0%)	7 (4.6%)	3 (2.7%)	11 (8.8%)	0 (0%)	0 (0%)	16 (18.4%)	10 (8%)	8 (7.3%)	4 (4.3%)	6 (8.6%)	4 (5.1%)
**Receipt of any HIV/STI service plus...**												
Family Planning Provision	0 (0%)	1 (.7%)	0 (0%)	2 (1.6%)	0 (0%)	0 (0%)	5 (5.7%)	9 (7.2%)	2 (1.8%)	1 (1.1%)	4 (5.7%)	1 (1.3%)
Family Planning Counselling	0 (0%)	1 (.7%)	1 (.9%)	1 (.8%)	0 (0%)	0 (0%)	3 (3.4%)	2 (1.6%)	1 (.9%)	1 (1.1%)	2 (2.9%)	2 (2.5%)
ANC	0 (0%)	6 (3.9%)	3 (2.7%)	9 (7.2%)	0 (0%)	0 (0%)	11 (12.6%)	0 (0%)	5 (4.6%)	3 (3.2%)	0 (0%)	3 (3.8%)
PNC (mother or baby)	0 (0%)	0 (0%)	0 (0%)	0 (0%)	0 (0%)	0 (0%)	1 (1.1%)	1 (.8%)	1 (.9%)	0 (0%)	0 (0%)	0 (0%)
cervical screening	0 (0%)	0 (0%)	0 (0%)	0 (0%)	0 (0%)	0 (0%)	0 (0%)	0 (0%)	0 (0%)	0 (0%)	0 (0%)	0 (0%)
child welfare (incl. Immunization)	0 (0%)	1 (.7%)	1 (.9%)	0 (0%)	0 (0%)	0 (0%)	1 (1.1%)	2 (1.6%)	4 (3.7%)	1 (1.1%)	3 (4.3%)	1 (1.3%)
Other RH/MCH service	0 (0%)	0 (0%)	0 (0%)	0 (0%)	0 (0%)	0 (0%)	0 (0%)	0 (0%)	0 (0%)	0 (0%)	0 (0%)	0 (0%)

	Facility K						Facility L					
	Jun2009	Jan2010	Jun2010	Jan2011	Jun2011	Jan2012	Jun2009	Jan2010	Jun2010	Jan2011	Jun2011	Jan2012
**Visits tracked**	221	146	117	247	172	141	107	125	38	52	46	62
**Client age (years)**												
Mean [sd]	27.3 [6.8]	29.2 [7.6]	29.2 [6.4]	29.1 [8]	26.5 [8.2]	27.8 [8.9]	27.6 [6.8]	28.5 [8.1]	26.1 [5.9]	25.4 [6.1]	26.1 [7.2]	28.4 [5.4]
Missing	0 (0%)	1 (.7%)	0 (0%)	3 (1.2%)	1 (.6%)	0 (0%)	0 (0%)	1 (.8%)	0 (0%)	0 (0%)	0 (0%)	4 (6.5%)
**Services received per client**												
none	0 (0%)	0 (0%)	0 (0%)	0 (0%)	1 (.6%)	0 (0%)	0 (0%)	0 (0%)	0 (0%)	0 (0%)	0 (0%)	1 (1.6%)
one	157 (71%)	69 (47.3%)	92 (78.6%)	164 (66.4%)	83 (48.3%)	95 (67.4%)	24 (22.4%)	6 (4.8%)	36 (94.7%)	30 (57.7%)	22 (47.8%)	30 (48.4%)
two or more	64 (29%)	77 (52.7%)	25 (21.4%)	83 (33.6%)	88 (51.2%)	46 (32.6%)	83 (77.6%)	119 (95.2%)	2 (5.3%)	22 (42.3%)	24 (52.2%)	31 (50%)
**Visits where at least one services was received...**												
Mean [sd] services received	1.3 [.5]	1.6 [.7]	1.3 [.9]	1.7 [1]	1.8 [1]	1.4 [.5]	2.3 [1]	3.6 [1.5]	1.1 [.4]	1.6 [.8]	1.6 [.7]	1.6 [.6]
Mean [sd] providers seen	1 [0]	1.3 [.6]	1 [.3]	1.4 [.8]	1.1 [.2]	1 [.2]	1 [.1]	1.6 [.9]	1 [0]	1 [0]	1 [0]	1 [0]
**Top 3 services provided**												
1	Child welfare	Child welfare	Child welfare	Child welfare	Child welfare	Child welfare	Child welfare	Child welfare	Child welfare	Child welfare	Child welfare	Child welfare
2	ANC	Other services	ANC	Other services	general counsel	injection	FP counselling	general counsel	ANC	ANC	ANC	ANC
3	FP provision	FP provision	FP provision	pharmacy	ANC	Other services	PNC	pharmacy	FP provision	HIV counselling	Other services	general counselling
**HIV/STI services received**												
HIV testing	0 (0%)	0 (0%)	2 (1.7%)	0 (0%)	10 (5.8%)	0 (0%)	10 (9.3%)	4 (3.2%)	1 (2.6%)	9 (17.3%)	1 (2.2%)	0 (0%)
HIV counselling	0 (0%)	0 (0%)	2 (1.7%)	0 (0%)	11 (6.4%)	1 (.7%)	12 (11.2%)	5 (4%)	0 (0%)	9 (17.3%)	1 (2.2%)	0 (0%)
HIV treatment	0 (0%)	0 (0%)	0 (0%)	2 (.8%)	0 (0%)	0 (0%)	0 (0%)	0 (0%)	0 (0%)	0 (0%)	0 (0%)	0 (0%)
HIV PMTCT	0 (0%)	0 (0%)	0 (0%)	0 (0%)	11 (6.4%)	0 (0%)	0 (0%)	6 (4.8%)	0 (0%)	0 (0%)	0 (0%)	0 (0%)
Other HIV/STI service	0 (0%)	0 (0%)	0 (0%)	0 (0%)	0 (0%)	0 (0%)	0 (0%)	0 (0%)	0 (0%)	0 (0%)	0 (0%)	0 (0%)
STI counselling or testing	0 (0%)	0 (0%)	0 (0%)	0 (0%)	0 (0%)	0 (0%)	0 (0%)	0 (0%)	0 (0%)	0 (0%)	0 (0%)	0 (0%)
Any HIV/STI services	0 (0%)	0 (0%)	2 (1.7%)	2 (.8%)	11 (6.4%)	1 (.7%)	12 (11.2%)	5 (4%)	1 (2.6%)	10 (19.2%)	1 (2.2%)	0 (0%)
**MCH services received**												
Family Planning Provision	21 (9.5%)	18 (12.3%)	6 (5.1%)	25 (10.1%)	13 (7.6%)	3 (2.1%)	4 (3.7%)	4 (3.2%)	3 (7.9%)	5 (9.6%)	2 (4.3%)	4 (6.5%)
Family Planning Counselling	18 (8.1%)	9 (6.2%)	3 (2.6%)	2 (.8%)	0 (0%)	3 (2.1%)	68 (63.6%)	32 (25.6%)	1 (2.6%)	1 (1.9%)	0 (0%)	3 (4.8%)
ANC	35 (15.8%)	4 (2.7%)	13 (11.1%)	26 (10.5%)	29 (16.9%)	8 (5.7%)	14 (13.1%)	9 (7.2%)	13 (34.2%)	26 (50%)	13 (28.3%)	8 (12.9%)
PNC (mother or baby)	1 (.5%)	0 (0%)	4 (3.4%)	9 (3.6%)	1 (.6%)	0 (0%)	9 (8.4%)	1 (.8%)	0 (0%)	0 (0%)	0 (0%)	0 (0%)
cervical screening	0 (0%)	0 (0%)	0 (0%)	0 (0%)	0 (0%)	0 (0%)	0 (0%)	0 (0%)	0 (0%)	0 (0%)	0 (0%)	0 (0%)
child welfare (incl. Immunization)	136 (61.5%)	126 (86.3%)	99 (84.6%)	192 (77.7%)	109 (63.4%)	116 (82.3%)	91 (85%)	113 (90.4%)	22 (57.9%)	26 (50%)	31 (67.4%)	53 (85.5%)
Other	0 (0%)	0 (0%)	0 (0%)	0 (0%)	0 (0%)	0 (0%)	0 (0%)	0 (0%)	0 (0%)	0 (0%)	0 (0%)	0 (0%)
Any MCH service	74 (33.5%)	22 (15.1%)	23 (19.7%)	62 (25.1%)	43 (25%)	12 (8.5%)	71 (66.4%)	36 (28.8%)	16 (42.1%)	29 (55.8%)	15 (32.6%)	14 (22.6%)
**Integrated HIV/STI & MCH services received**												
neither	147 (66.5%)	124 (84.9%)	94 (80.3%)	184 (74.5%)	121 (70.3%)	129 (91.5%)	36 (33.6%)	89 (71.2%)	22 (57.9%)	21 (40.4%)	31 (67.4%)	48 (77.4%)
MCH only	74 (33.5%)	22 (15.1%)	21 (17.9%)	61 (24.7%)	40 (23.3%)	11 (7.8%)	59 (55.1%)	31 (24.8%)	15 (39.5%)	21 (40.4%)	14 (30.4%)	14 (22.6%)
HIV/STI only	0 (0%)	0 (0%)	0 (0%)	1 (.4%)	8 (4.7%)	0 (0%)	0 (0%)	0 (0%)	0 (0%)	2 (3.8%)	0 (0%)	0 (0%)
HIV/STI & MCH	0 (0%)	0 (0%)	2 (1.7%)	1 (.4%)	3 (1.7%)	1 (.7%)	12 (11.2%)	5 (4%)	1 (2.6%)	8 (15.4%)	1 (2.2%)	0 (0%)
**Receipt of any HIV/STI service plus...**												
Family Planning Provision	0 (0%)	0 (0%)	0 (0%)	0 (0%)	1 (.6%)	0 (0%)	2 (1.9%)	0 (0%)	0 (0%)	0 (0%)	0 (0%)	0 (0%)
Family Planning Counselling	0 (0%)	0 (0%)	0 (0%)	0 (0%)	0 (0%)	0 (0%)	9 (8.4%)	4 (3.2%)	1 (2.6%)	0 (0%)	0 (0%)	0 (0%)
ANC	0 (0%)	0 (0%)	1 (.9%)	1 (.4%)	1 (.6%)	1 (.7%)	10 (9.3%)	5 (4%)	1 (2.6%)	8 (15.4%)	1 (2.2%)	0 (0%)
PNC (mother or baby)	0 (0%)	0 (0%)	1 (.9%)	0 (0%)	1 (.6%)	0 (0%)	0 (0%)	0 (0%)	0 (0%)	0 (0%)	0 (0%)	0 (0%)
cervical screening	0 (0%)	0 (0%)	0 (0%)	0 (0%)	0 (0%)	0 (0%)	0 (0%)	0 (0%)	0 (0%)	0 (0%)	0 (0%)	0 (0%)
child welfare (incl. Immunization)	0 (0%)	0 (0%)	1 (.9%)	0 (0%)	8 (4.7%)	0 (0%)	0 (0%)	0 (0%)	0 (0%)	1 (1.9%)	0 (0%)	0 (0%)
Other RH/MCH service	0 (0%)	0 (0%)	0 (0%)	0 (0%)	0 (0%)	0 (0%)	0 (0%)	0 (0%)	0 (0%)	0 (0%)	0 (0%)	0 (0%)
