# Supplementary material for: Integration of HIV and reproductive health services in public sector facilities: analysis of client flow data over time in Kenya
Source: BMJ Glob Health. 2018 Sep 14;3(5):e000867. doi: 10.1136/bmjgh-2018-000867 (PMC6144905; doi:10.1136/bmjgh-2018-000867)
Supplement: Supplementary file 5 [file bmjgh-2018-000867supp005.pdf]

Supplemental Table 4. Characteristics of client visits by facility, round and service type in Central Province (HIV-FP)

	District C1 Facilities: Facility a						Facility b					
	Jun2009	Jan2010	Jun2010	Jan2011	Jun2011	Jan2012	Jun2009	Jan2010	Jun2010	Jan2011	Jun2011	Jan2012
**Visits tracked**	326	236	280	403	358	581	318	259	250	320	303	327
**Client age (years)**												
Mean [sd]	26.2 [5.7]	25.4 [5.4]	25.9 [5.9]	25.6 [4.8]	25.8 [5.1]	25.8 [5.6]	26.5 [6.2]	27.3 [5.8]	27.1 [7.5]	26.7 [5.2]	27.4 [6]	28.6 [6.2]
Missing	0 (0%)	8 (3.4%)	1 (.4%)	9 (2.2%)	5 (1.4%)	5 (.9%)	0 (0%)	9 (3.5%)	0 (0%)	6 (1.9%)	15 (5%)	6 (1.8%)
**Services received per client**												
none	0 (0%)	4 (1.7%)	1 (.4%)	2 (.5%)	1 (.3%)	0 (0%)	0 (0%)	2 (.8%)	0 (0%)	0 (0%)	1 (.3%)	3 (.9%)
one	120 (36.8%)	103 (43.6%)	145 (51.8%)	113 (28%)	138 (38.5%)	307 (52.8%)	107 (33.6%)	98 (37.8%)	144 (57.6%)	165 (51.6%)	94 (31%)	75 (22.9%)
two or more	206 (63.2%)	129 (54.7%)	134 (47.9%)	288 (71.5%)	219 (61.2%)	274 (47.2%)	211 (66.4%)	159 (61.4%)	106 (42.4%)	155 (48.4%)	208 (68.6%)	249 (76.1%)
**Visits where at least one services was received...**												
Mean [sd] services received	2.1 [1.3]	2.1 [1.2]	1.7 [1.1]	2 [.8]	2.2 [1.4]	1.9 [1.2]	2.4 [1.5]	2.2 [1.4]	1.7 [1]	1.8 [1.2]	2 [.9]	2.6 [1.7]
Mean [sd] providers seen	2 [1]	1.1 [.2]	1.2 [.5]	1.3 [.5]	1.3 [.6]	1.4 [.7]	1.2 [.5]	1.1 [.3]	1.2 [.4]	1.1 [.4]	1.3 [.6]	1.6 [.8]
**Top 3 services provided**												
1	Child welfare	Child welfare	Child welfare	Child welfare	Child welfare	ANC	Child welfare	Child welfare	Child welfare	Child welfare	Child welfare	Child welfare
2	ANC	ANC	ANC	ANC	ANC	Child welfare	ANC	ANC	ANC	FP provision	ANC	ANC
3	FP provision	general counselling	FP provision	FP provision	FP provision	FP provision	FP counselling	HIV counselling	FP provision	ANC	FP provision	general counselling
**HIV/STI services received**												
HIV testing	12 (3.7%)	31 (13.1%)	17 (6.1%)	0 (0%)	17 (4.7%)	52 (9%)	42 (13.2%)	19 (7.3%)	5 (2%)	33 (10.3%)	7 (2.3%)	16 (4.9%)
HIV counselling	19 (5.8%)	46 (19.5%)	18 (6.4%)	0 (0%)	18 (5%)	47 (8.1%)	72 (22.6%)	56 (21.6%)	5 (2%)	34 (10.6%)	11 (3.6%)	17 (5.2%)
HIV treatment	34 (10.4%)	15 (6.4%)	2 (.7%)	1 (.2%)	0 (0%)	1 (.2%)	5 (1.6%)	10 (3.9%)	8 (3.2%)	13 (4.1%)	0 (0%)	2 (.6%)
HIV PMTCT	0 (0%)	9 (3.8%)	14 (5%)	30 (7.4%)	3 (.8%)	56 (9.6%)	0 (0%)	20 (7.7%)	28 (11.2%)	8 (2.5%)	20 (6.6%)	54 (16.5%)
Other HIV/STI service	0 (0%)	0 (0%)	0 (0%)	0 (0%)	0 (0%)	0 (0%)	0 (0%)	0 (0%)	0 (0%)	0 (0%)	0 (0%)	0 (0%)
STI counselling or testing	6 (1.8%)	0 (0%)	3 (1.1%)	4 (1%)	5 (1.4%)	0 (0%)	59 (18.6%)	34 (13.1%)	8 (3.2%)	1 (.3%)	3 (1%)	8 (2.4%)
Any HIV/STI services	45 (13.8%)	47 (19.9%)	21 (7.5%)	5 (1.2%)	25 (7%)	54 (9.3%)	84 (26.4%)	75 (29%)	22 (8.8%)	51 (15.9%)	16 (5.3%)	22 (6.7%)
**MCH services received**												
Family Planning Provision	55 (16.9%)	24 (10.2%)	36 (12.9%)	63 (15.6%)	84 (23.5%)	142 (24.4%)	79 (24.8%)	38 (14.7%)	47 (18.8%)	74 (23.1%)	78 (25.7%)	56 (17.1%)
Family Planning Counselling	24 (7.4%)	44 (18.6%)	22 (7.9%)	36 (8.9%)	65 (18.2%)	32 (5.5%)	81 (25.5%)	34 (13.1%)	16 (6.4%)	63 (19.7%)	35 (11.6%)	62 (19%)
ANC	139 (42.6%)	106 (44.9%)	93 (33.2%)	103 (25.6%)	133 (37.2%)	272 (46.8%)	85 (26.7%)	115 (44.4%)	94 (37.6%)	74 (23.1%)	93 (30.7%)	120 (36.7%)
PNC (mother or baby)	23 (7.1%)	3 (1.3%)	1 (.4%)	4 (1%)	3 (.8%)	3 (.5%)	4 (1.3%)	7 (2.7%)	10 (4%)	15 (4.7%)	19 (6.3%)	5 (1.5%)
cervical screening	9 (2.8%)	0 (0%)	0 (0%)	0 (0%)	1 (.3%)	3 (.5%)	21 (6.6%)	1 (.4%)	11 (4.4%)	1 (.3%)	8 (2.6%)	9 (2.8%)
child welfare (incl. Immunization)	198 (60.7%)	65 (27.5%)	152 (54.3%)	225 (55.8%)	194 (54.2%)	224 (38.6%)	157 (49.4%)	89 (34.4%)	104 (41.6%)	145 (45.3%)	118 (38.9%)	130 (39.8%)
Other	0 (0%)	1 (.4%)	2 (.7%)	0 (0%)	1 (.3%)	3 (.5%)	0 (0%)	0 (0%)	0 (0%)	3 (.9%)	6 (2%)	2 (.6%)
Any MCH service	185 (56.7%)	152 (64.4%)	131 (46.8%)	176 (43.7%)	220 (61.5%)	376 (64.7%)	182 (57.2%)	162 (62.5%)	160 (64%)	164 (51.2%)	194 (64%)	169 (51.7%)
**Integrated HIV/STI & MCH services received**												
neither	121 (37.1%)	66 (28%)	149 (53.2%)	226 (56.1%)	138 (38.5%)	203 (34.9%)	130 (40.9%)	88 (34%)	83 (33.2%)	138 (43.1%)	108 (35.6%)	153 (46.8%)
MCH only	160 (49.1%)	123 (52.1%)	110 (39.3%)	172 (42.7%)	195 (54.5%)	324 (55.8%)	104 (32.7%)	96 (37.1%)	145 (58%)	131 (40.9%)	179 (59.1%)	152 (46.5%)
HIV/STI only	20 (6.1%)	18 (7.6%)	0 (0%)	1 (.2%)	0 (0%)	2 (.3%)	6 (1.9%)	9 (3.5%)	7 (2.8%)	18 (5.6%)	1 (.3%)	5 (1.5%)
HIV/STI & MCH	25 (7.7%)	29 (12.3%)	21 (7.5%)	4 (1%)	25 (7%)	52 (9%)	78 (24.5%)	66 (25.5%)	15 (6%)	33 (10.3%)	15 (5%)	17 (5.2%)
**Receipt of any HIV/STI service plus...**												
Family Planning Provision	2 (.6%)	7 (3%)	1 (.4%)	2 (.5%)	20 (5.6%)	36 (6.2%)	59 (18.6%)	11 (4.2%)	7 (2.8%)	16 (5%)	7 (2.3%)	3 (.9%)
Family Planning Counselling	1 (.3%)	26 (11%)	1 (.4%)	3 (.7%)	18 (5%)	17 (2.9%)	59 (18.6%)	12 (4.6%)	7 (2.8%)	13 (4.1%)	1 (.3%)	15 (4.6%)
ANC	19 (5.8%)	3 (1.3%)	20 (7.1%)	1 (.2%)	5 (1.4%)	28 (4.8%)	16 (5%)	55 (21.2%)	6 (2.4%)	13 (4.1%)	9 (3%)	15 (4.6%)
PNC (mother or baby)	5 (1.5%)	1 (.4%)	0 (0%)	0 (0%)	0 (0%)	0 (0%)	0 (0%)	2 (.8%)	2 (.8%)	9 (2.8%)	1 (.3%)	0 (0%)
cervical screening	1 (.3%)	0 (0%)	0 (0%)	0 (0%)	0 (0%)	1 (.2%)	9 (2.8%)	0 (0%)	2 (.8%)	0 (0%)	1 (.3%)	0 (0%)
child welfare (incl. Immunization)	24 (7.4%)	4 (1.7%)	0 (0%)	2 (.5%)	12 (3.4%)	10 (1.7%)	12 (3.8%)	3 (1.2%)	6 (2.4%)	4 (1.3%)	0 (0%)	0 (0%)
Other RH/MCH service	0 (0%)	0 (0%)	1 (.4%)	0 (0%)	0 (0%)	1 (.2%)	0 (0%)	0 (0%)	0 (0%)	0 (0%)	1 (.3%)	0 (0%)

	District C1 Facilities: Facility c						Facility d					
	Jun2009	Jan2010	Jun2010	Jan2011	Jun2011	Jan2012	Jun2009	Jan2010	Jun2010	Jan2011	Jun2011	Jan2012
**Visits tracked**	190	232	224	268	257	365	277	235	194	200	174	152
**Client age (years)**												
Mean [sd]	24.7 [4.9]	24.9 [5.1]	24.8 [5.4]	26.4 [5.4]	25.8 [5]	25.3 [5.3]	30.7 [12.1]	27 [6.2]	27.1 [5.9]	27.5 [6.1]	27.4 [6.7]	28.3 [7.6]
Missing	0 (0%)	4 (1.7%)	0 (0%)	9 (3.4%)	1 (.4%)	3 (.8%)	0 (0%)	1 (.4%)	0 (0%)	2 (1%)	2 (1.1%)	1 (.7%)
**Services received per client**												
none	2 (1.1%)	1 (.4%)	0 (0%)	0 (0%)	0 (0%)	0 (0%)	8 (2.9%)	1 (.4%)	0 (0%)	0 (0%)	1 (.6%)	0 (0%)
one	73 (38.4%)	99 (42.7%)	107 (47.8%)	110 (41%)	104 (40.5%)	145 (39.7%)	123 (44.4%)	99 (42.1%)	103 (53.1%)	99 (49.5%)	64 (36.8%)	60 (39.5%)
two or more	115 (60.5%)	132 (56.9%)	117 (52.2%)	158 (59%)	153 (59.5%)	220 (60.3%)	146 (52.7%)	135 (57.4%)	91 (46.9%)	101 (50.5%)	109 (62.6%)	92 (60.5%)
**Visits where at least one services was received...**												
Mean [sd] services received	1.9 [.9]	2.1 [1.2]	2.2 [1.6]	2 [1.4]	2.2 [1.4]	2.1 [1.3]	1.8 [.9]	2.1 [1.2]	1.6 [.7]	1.5 [.6]	1.7 [.7]	1.8 [.9]
Mean [sd] providers seen	1.1 [.4]	1 [.2]	1.2 [.5]	1.2 [.4]	1.4 [.8]	1.2 [.5]	1.2 [.4]	1 [.2]	1.1 [.4]	1 [.2]	1.2 [.4]	1.1 [.4]
**Top 3 services provided**												
1	Child welfare	Child welfare	Child welfare	Child welfare	Child welfare	Child welfare	Child welfare	Child welfare	Child welfare	Child welfare	Child welfare	Child welfare
2	ANC	HIV counsellin	ANC	ANC	ANC	ANC	FP provision	FP provision	FP provision	FP provision	ANC	FP provision
3	FP counselling	ANC	HIV counselling	Other services	FP provision	general counselling	FP counselling	FP counselling	ANC	FP counselling	FP provision	FP counselling
**HIV/STI services received**												
HIV testing	19 (10%)	53 (22.8%)	30 (13.4%)	16 (6%)	16 (6.2%)	14 (3.8%)	25 (9%)	14 (6%)	0 (0%)	2 (1%)	0 (0%)	0 (0%)
HIV counselling	19 (10%)	57 (24.6%)	32 (14.3%)	16 (6%)	16 (6.2%)	15 (4.1%)	13 (4.7%)	18 (7.7%)	1 (.5%)	0 (0%)	1 (.6%)	0 (0%)
HIV treatment	0 (0%)	7 (3%)	2 (.9%)	1 (.4%)	5 (1.9%)	2 (.5%)	32 (11.6%)	0 (0%)	3 (1.5%)	0 (0%)	4 (2.3%)	0 (0%)
HIV PMTCT	0 (0%)	29 (12.5%)	29 (12.9%)	6 (2.2%)	4 (1.6%)	13 (3.6%)	0 (0%)	40 (17%)	2 (1%)	3 (1.5%)	8 (4.6%)	0 (0%)
Other HIV/STI service	0 (0%)	0 (0%)	0 (0%)	0 (0%)	0 (0%)	0 (0%)	0 (0%)	0 (0%)	0 (0%)	0 (0%)	0 (0%)	0 (0%)
STI counselling or testing	4 (2.1%)	1 (.4%)	0 (0%)	8 (3%)	1 (.4%)	0 (0%)	0 (0%)	18 (7.7%)	1 (.5%)	0 (0%)	0 (0%)	0 (0%)
Any HIV/STI services	24 (12.6%)	61 (26.3%)	33 (14.7%)	17 (6.3%)	23 (8.9%)	16 (4.4%)	56 (20.2%)	40 (17%)	5 (2.6%)	2 (1%)	4 (2.3%)	0 (0%)
**MCH services received**												
Family Planning Provision	23 (12.1%)	52 (22.4%)	25 (11.2%)	26 (9.7%)	75 (29.2%)	36 (9.9%)	65 (23.5%)	76 (32.3%)	79 (40.7%)	99 (49.5%)	36 (20.7%)	48 (31.6%)
Family Planning Counselling	30 (15.8%)	54 (23.3%)	15 (6.7%)	24 (9%)	38 (14.8%)	52 (14.2%)	52 (18.8%)	50 (21.3%)	11 (5.7%)	31 (15.5%)	34 (19.5%)	45 (29.6%)
ANC	80 (42.1%)	54 (23.3%)	86 (38.4%)	66 (24.6%)	74 (28.8%)	140 (38.4%)	48 (17.3%)	38 (16.2%)	38 (19.6%)	25 (12.5%)	42 (24.1%)	45 (29.6%)
PNC (mother or baby)	3 (1.6%)	0 (0%)	12 (5.4%)	17 (6.3%)	23 (8.9%)	27 (7.4%)	30 (10.8%)	40 (17%)	2 (1%)	2 (1%)	1 (.6%)	1 (.7%)
cervical screening	4 (2.1%)	4 (1.7%)	1 (.4%)	0 (0%)	2 (.8%)	3 (.8%)	2 (.7%)	2 (.9%)	1 (.5%)	0 (0%)	0 (0%)	0 (0%)
child welfare (incl. Immunization)	82 (43.2%)	102 (44%)	125 (55.8%)	134 (50%)	119 (46.3%)	171 (46.8%)	119 (43%)	106 (45.1%)	89 (45.9%)	76 (38%)	96 (55.2%)	66 (43.4%)
Other	0 (0%)	1 (.4%)	0 (0%)	1 (.4%)	3 (1.2%)	0 (0%)	3 (1.1%)	0 (0%)	0 (0%)	0 (0%)	2 (1.1%)	0 (0%)
Any MCH service	111 (58.4%)	109 (47%)	121 (54%)	106 (39.6%)	168 (65.4%)	203 (55.6%)	138 (49.8%)	154 (65.5%)	115 (59.3%)	127 (63.5%)	81 (46.6%)	94 (61.8%)
**Integrated HIV/STI & MCH services received**												
neither	68 (35.8%)	107 (46.1%)	100 (44.6%)	162 (60.4%)	84 (32.7%)	160 (43.8%)	101 (36.5%)	80 (34%)	77 (39.7%)	73 (36.5%)	89 (51.1%)	58 (38.2%)
MCH only	98 (51.6%)	64 (27.6%)	91 (40.6%)	89 (33.2%)	150 (58.4%)	189 (51.8%)	120 (43.3%)	115 (48.9%)	112 (57.7%)	125 (62.5%)	81 (46.6%)	94 (61.8%)
HIV/STI only	11 (5.8%)	16 (6.9%)	3 (1.3%)	0 (0%)	5 (1.9%)	2 (.5%)	38 (13.7%)	1 (.4%)	2 (1%)	0 (0%)	4 (2.3%)	0 (0%)
HIV/STI & MCH	13 (6.8%)	45 (19.4%)	30 (13.4%)	17 (6.3%)	18 (7%)	14 (3.8%)	18 (6.5%)	39 (16.6%)	3 (1.5%)	2 (1%)	0 (0%)	0 (0%)
**Receipt of any HIV/STI service plus...**												
Family Planning Provision	2 (1.1%)	41 (17.7%)	6 (2.7%)	9 (3.4%)	7 (2.7%)	2 (.5%)	2 (.7%)	26 (11.1%)	2 (1%)	1 (.5%)	0 (0%)	0 (0%)
Family Planning Counselling	3 (1.6%)	44 (19%)	6 (2.7%)	8 (3%)	1 (.4%)	4 (1.1%)	3 (1.1%)	26 (11.1%)	0 (0%)	0 (0%)	0 (0%)	0 (0%)
ANC	10 (5.3%)	1 (.4%)	20 (8.9%)	1 (.4%)	8 (3.1%)	9 (2.5%)	12 (4.3%)	12 (5.1%)	1 (.5%)	1 (.5%)	0 (0%)	0 (0%)
PNC (mother or baby)	3 (1.6%)	0 (0%)	3 (1.3%)	10 (3.7%)	4 (1.6%)	5 (1.4%)	3 (1.1%)	2 (.9%)	0 (0%)	0 (0%)	0 (0%)	0 (0%)
cervical screening	0 (0%)	3 (1.3%)	1 (.4%)	0 (0%)	2 (.8%)	0 (0%)	1 (.4%)	1 (.4%)	0 (0%)	0 (0%)	0 (0%)	0 (0%)
child welfare (incl. Immunization)	9 (4.7%)	3 (1.3%)	7 (3.1%)	12 (4.5%)	7 (2.7%)	5 (1.4%)	6 (2.2%)	0 (0%)	2 (1%)	0 (0%)	3 (1.7%)	0 (0%)
Other RH/MCH service	0 (0%)	1 (.4%)	0 (0%)	1 (.4%)	2 (.8%)	0 (0%)	0 (0%)	0 (0%)	0 (0%)	0 (0%)	0 (0%)	0 (0%)

	District C1 Facilities: Facility e						Facility f					
	Jun2009	Jan2010	Jun2010	Jan2011	Jun2011	Jan2012	Jun2009	Jan2010	Jun2010	Jan2011	Jun2011	Jan2012
**Visits tracked**	250	120	103	108	74	58	280	126	138	117	126	48
**Client age (years)**												
Mean [sd]	30.4 [13.6]	28 [7]	28.1 [7.5]	28.4 [10.1]	28.7 [7.5]	30 [9.5]	33.8 [14.2]	29.1 [6.2]	29.8 [7.8]	29.3 [7.3]	26.9 [4.3]	29.9 [7.4]
Missing	0 (0%)	7 (5.8%)	0 (0%)	0 (0%)	1 (1.4%)	0 (0%)	0 (0%)	1 (.8%)	0 (0%)	0 (0%)	0 (0%)	1 (2.1%)
**Services received per client**												
none	0 (0%)	1 (.8%)	0 (0%)	0 (0%)	0 (0%)	0 (0%)	2 (.7%)	1 (.8%)	0 (0%)	1 (.9%)	0 (0%)	0 (0%)
one	151 (60.4%)	47 (39.2%)	15 (14.6%)	41 (38%)	14 (18.9%)	10 (17.2%)	69 (24.6%)	31 (24.6%)	34 (24.6%)	34 (29.1%)	126 (100%)	36 (75%)
two or more	99 (39.6%)	72 (60%)	88 (85.4%)	67 (62%)	60 (81.1%)	48 (82.8%)	209 (74.6%)	94 (74.6%)	104 (75.4%)	82 (70.1%)	0 (0%)	12 (25%)
**Visits where at least one services was received...**												
Mean [sd] services received	1.6 [.9]	2 [1.1]	3.2 [1.4]	2 [1.1]	2.8 [1.3]	2.5 [1.1]	2.3 [1.3]	2.5 [1.5]	2.6 [1.3]	2.1 [.9]	1 [0]	1.3 [.5]
Mean [sd] providers seen	1.1 [.3]	1 [.1]	1.5 [.5]	1.2 [.4]	1.1 [.3]	1.4 [.7]	1.7 [.8]	1.4 [.6]	1.6 [.8]	1.3 [.6]	1 [0]	1.1 [.3]
**Top 3 services provided**												
1	Other services	Child welfare	Child welfare	Child welfare	Child welfare	Child welfare	Other services	Child welfare	Child welfare	Child welfare	Child welfare	Child welfare
2	Child welfare	FP provision	HIV counselling	FP provision	HIV counselling	FP provision	pharmacy	FP provision	Other services	pharmacy	ANC	ANC
3	FP counselling	FP counselling	HIV testing	FP counselling	HIV testing	FP counselling	Child welfare	pharmacy	pharmacy	Other services	HIV counselling	FP provision
**HIV/STI services received**												
HIV testing	28 (11.2%)	17 (14.2%)	47 (45.6%)	8 (7.4%)	39 (52.7%)	10 (17.2%)	27 (9.6%)	22 (17.5%)	19 (13.8%)	4 (3.4%)	6 (4.8%)	0 (0%)
HIV counselling	7 (2.8%)	13 (10.8%)	57 (55.3%)	4 (3.7%)	39 (52.7%)	14 (24.1%)	40 (14.3%)	27 (21.4%)	19 (13.8%)	8 (6.8%)	7 (5.6%)	0 (0%)
HIV treatment	0 (0%)	1 (.8%)	7 (6.8%)	0 (0%)	0 (0%)	2 (3.4%)	4 (1.4%)	1 (.8%)	1 (.7%)	0 (0%)	6 (4.8%)	0 (0%)
HIV PMTCT	1 (.4%)	2 (1.7%)	0 (0%)	0 (0%)	3 (4.1%)	1 (1.7%)	0 (0%)	4 (3.2%)	3 (2.2%)	2 (1.7%)	0 (0%)	0 (0%)
Other HIV/STI service	0 (0%)	0 (0%)	0 (0%)	0 (0%)	0 (0%)	0 (0%)	0 (0%)	0 (0%)	0 (0%)	0 (0%)	0 (0%)	0 (0%)
STI counselling or testing	1 (.4%)	0 (0%)	14 (13.6%)	0 (0%)	1 (1.4%)	2 (3.4%)	25 (8.9%)	7 (5.6%)	2 (1.4%)	1 (.9%)	0 (0%)	0 (0%)
Any HIV/STI services	34 (13.6%)	21 (17.5%)	68 (66%)	9 (8.3%)	39 (52.7%)	17 (29.3%)	44 (15.7%)	27 (21.4%)	22 (15.9%)	9 (7.7%)	19 (15.1%)	0 (0%)
**MCH services received**												
Family Planning Provision	36 (14.4%)	47 (39.2%)	34 (33%)	46 (42.6%)	29 (39.2%)	18 (31%)	33 (11.8%)	40 (31.7%)	31 (22.5%)	19 (16.2%)	5 (4%)	8 (16.7%)
Family Planning Counselling	40 (16%)	25 (20.8%)	48 (46.6%)	24 (22.2%)	13 (17.6%)	16 (27.6%)	17 (6.1%)	34 (27%)	23 (16.7%)	12 (10.3%)	4 (3.2%)	3 (6.3%)
ANC	7 (2.8%)	9 (7.5%)	23 (22.3%)	2 (1.9%)	12 (16.2%)	6 (10.3%)	25 (8.9%)	18 (14.3%)	21 (15.2%)	25 (21.4%)	13 (10.3%)	8 (16.7%)
PNC (mother or baby)	5 (2%)	2 (1.7%)	1 (1%)	11 (10.2%)	3 (4.1%)	2 (3.4%)	0 (0%)	2 (1.6%)	0 (0%)	0 (0%)	0 (0%)	0 (0%)
cervical screening	1 (.4%)	0 (0%)	0 (0%)	1 (.9%)	2 (2.7%)	2 (3.4%)	3 (1.1%)	0 (0%)	1 (.7%)	0 (0%)	0 (0%)	1 (2.1%)
child welfare (incl. Immunization)	63 (25.2%)	51 (42.5%)	48 (46.6%)	62 (57.4%)	32 (43.2%)	32 (55.2%)	61 (21.8%)	87 (69%)	62 (44.9%)	55 (47%)	85 (67.5%)	32 (66.7%)
Other	0 (0%)	0 (0%)	0 (0%)	0 (0%)	0 (0%)	0 (0%)	7 (2.5%)	0 (0%)	3 (2.2%)	0 (0%)	0 (0%)	0 (0%)
Any MCH service	55 (22%)	60 (50%)	66 (64.1%)	59 (54.6%)	44 (59.5%)	31 (53.4%)	62 (22.1%)	55 (43.7%)	55 (39.9%)	45 (38.5%)	22 (17.5%)	18 (37.5%)
**Integrated HIV/STI & MCH services received**												
neither	178 (71.2%)	58 (48.3%)	19 (18.4%)	49 (45.4%)	20 (27%)	27 (46.6%)	208 (74.3%)	70 (55.6%)	75 (54.3%)	71 (60.7%)	85 (67.5%)	30 (62.5%)
MCH only	38 (15.2%)	41 (34.2%)	16 (15.5%)	50 (46.3%)	15 (20.3%)	14 (24.1%)	28 (10%)	29 (23%)	41 (29.7%)	37 (31.6%)	22 (17.5%)	18 (37.5%)
HIV/STI only	17 (6.8%)	2 (1.7%)	18 (17.5%)	0 (0%)	10 (13.5%)	0 (0%)	10 (3.6%)	1 (.8%)	8 (5.8%)	1 (.9%)	19 (15.1%)	0 (0%)
HIV/STI & MCH	17 (6.8%)	19 (15.8%)	50 (48.5%)	9 (8.3%)	29 (39.2%)	17 (29.3%)	34 (12.1%)	26 (20.6%)	14 (10.1%)	8 (6.8%)	0 (0%)	0 (0%)
**Receipt of any HIV/STI service plus...**												
Family Planning Provision	13 (5.2%)	18 (15%)	30 (29.1%)	9 (8.3%)	25 (33.8%)	12 (20.7%)	21 (7.5%)	23 (18.3%)	11 (8%)	4 (3.4%)	0 (0%)	0 (0%)
Family Planning Counselling	15 (6%)	15 (12.5%)	36 (35%)	4 (3.7%)	9 (12.2%)	11 (19%)	13 (4.6%)	23 (18.3%)	10 (7.2%)	4 (3.4%)	0 (0%)	0 (0%)
ANC	1 (.4%)	1 (.8%)	17 (16.5%)	0 (0%)	3 (4.1%)	1 (1.7%)	12 (4.3%)	3 (2.4%)	2 (1.4%)	3 (2.6%)	0 (0%)	0 (0%)
PNC (mother or baby)	0 (0%)	0 (0%)	1 (1%)	1 (.9%)	0 (0%)	1 (1.7%)	0 (0%)	2 (1.6%)	0 (0%)	0 (0%)	0 (0%)	0 (0%)
cervical screening	0 (0%)	0 (0%)	0 (0%)	0 (0%)	1 (1.4%)	1 (1.7%)	0 (0%)	0 (0%)	1 (.7%)	0 (0%)	0 (0%)	0 (0%)
child welfare (incl. Immunization)	0 (0%)	1 (.8%)	23 (22.3%)	4 (3.7%)	14 (18.9%)	2 (3.4%)	2 (.7%)	6 (4.8%)	5 (3.6%)	0 (0%)	0 (0%)	0 (0%)
Other RH/MCH service	0 (0%)	0 (0%)	0 (0%)	0 (0%)	0 (0%)	0 (0%)	1 (.4%)	0 (0%)	0 (0%)	0 (0%)	0 (0%)	0 (0%)

	District E2 Facilities: Facility g						Facility h					
	Jun2009	Jan2010	Jun2010	Jan2011	Jun2011	Jan2012	Jun2009	Jan2010	Jun2010	Jan2011	Jun2011	Jan2012
**Visits tracked**	191	257	240	138	372	283	218	251	235	265	301	255
**Client age (years)**												
Mean [sd]	27.4 [6.8]	26.4 [5.8]	27.3 [6.3]	28.3 [7.4]	27.4 [6.2]	27.6 [6.2]	27.4 [6.8]	27.5 [6.2]	27.2 [6.8]	27.8 [6.5]	27.4 [6.3]	27.4 [7]
Missing	0 (0%)	1 (.4%)	0 (0%)	1 (.7%)	2 (.5%)	0 (0%)	0 (0%)	0 (0%)	0 (0%)	2 (.8%)	0 (0%)	0 (0%)
**Services received per client**												
none	0 (0%)	2 (.8%)	0 (0%)	0 (0%)	1 (.3%)	1 (.4%)	2 (.9%)	0 (0%)	1 (.4%)	0 (0%)	0 (0%)	1 (.4%)
one	73 (38.2%)	138 (53.7%)	28 (11.7%)	38 (27.5%)	96 (25.8%)	95 (33.6%)	21 (9.6%)	18 (7.2%)	37 (15.7%)	20 (7.5%)	84 (27.9%)	35 (13.7%)
two or more	118 (61.8%)	117 (45.5%)	212 (88.3%)	100 (72.5%)	275 (73.9%)	187 (66.1%)	195 (89.4%)	233 (92.8%)	197 (83.8%)	245 (92.5%)	217 (72.1%)	219 (85.9%)
**Visits where at least one services was received...**												
Mean [sd] services received	1.9 [.9]	1.7 [1]	3 [1.4]	2.2 [1.1]	2.7 [1.6]	2 [.9]	2.9 [1.3]	2.5 [1]	2.4 [1.1]	3.7 [1.8]	1.8 [.6]	2.3 [.9]
Mean [sd] providers seen	1.2 [.5]	1.1 [.3]	1.8 [.6]	1.2 [.4]	1.8 [.8]	1.7 [.9]	2 [.7]	1.8 [.7]	1.9 [.6]	2.2 [.7]	1.8 [.6]	1.7 [.6]
**Top 3 services provided**												
1	Child welfare	Child welfare	Child welfare	Child welfare	Child welfare	Child welfare	Child welfare	ANC	Child welfare	Child welfare	Child welfare	Child welfare
2	ANC	FP provision	pharmacy	Other services	pharmacy	Other services	ANC	Child welfare	Other services	Other services	Other services	Other services
3	FP provision	FP counselling	Other services	ANC	ANC	pharmacy	Other services	Other services	pharmacy	pharmacy	ANC	ANC
**HIV/STI services received**												
HIV testing	11 (5.8%)	7 (2.7%)	34 (14.2%)	4 (2.9%)	35 (9.4%)	0 (0%)	20 (9.2%)	2 (.8%)	3 (1.3%)	18 (6.8%)	0 (0%)	0 (0%)
HIV counselling	24 (12.6%)	18 (7%)	12 (5%)	11 (8%)	29 (7.8%)	0 (0%)	37 (17%)	2 (.8%)	7 (3%)	21 (7.9%)	0 (0%)	0 (0%)
HIV treatment	3 (1.6%)	0 (0%)	1 (.4%)	0 (0%)	5 (1.3%)	0 (0%)	1 (.5%)	0 (0%)	2 (.9%)	2 (.8%)	0 (0%)	7 (2.7%)
HIV PMTCT	0 (0%)	23 (8.9%)	2 (.8%)	0 (0%)	11 (3%)	4 (1.4%)	0 (0%)	3 (1.2%)	1 (.4%)	4 (1.5%)	12 (4%)	10 (3.9%)
Other HIV/STI service	0 (0%)	0 (0%)	0 (0%)	0 (0%)	0 (0%)	0 (0%)	0 (0%)	0 (0%)	0 (0%)	0 (0%)	0 (0%)	0 (0%)
STI counselling or testing	6 (3.1%)	5 (1.9%)	0 (0%)	7 (5.1%)	0 (0%)	1 (.4%)	10 (4.6%)	0 (0%)	2 (.9%)	0 (0%)	0 (0%)	0 (0%)
Any HIV/STI services	27 (14.1%)	18 (7%)	37 (15.4%)	14 (10.1%)	40 (10.8%)	1 (.4%)	39 (17.9%)	3 (1.2%)	9 (3.8%)	27 (10.2%)	0 (0%)	7 (2.7%)
**MCH services received**												
Family Planning Provision	28 (14.7%)	52 (20.2%)	13 (5.4%)	8 (5.8%)	23 (6.2%)	25 (8.8%)	61 (28%)	65 (25.9%)	23 (9.8%)	28 (10.6%)	53 (17.6%)	38 (14.9%)
Family Planning Counselling	26 (13.6%)	48 (18.7%)	12 (5%)	5 (3.6%)	18 (4.8%)	4 (1.4%)	51 (23.4%)	44 (17.5%)	12 (5.1%)	26 (9.8%)	4 (1.3%)	40 (15.7%)
ANC	39 (20.4%)	44 (17.1%)	24 (10%)	30 (21.7%)	53 (14.2%)	36 (12.7%)	108 (49.5%)	110 (43.8%)	21 (8.9%)	39 (14.7%)	75 (24.9%)	61 (23.9%)
PNC (mother or baby)	6 (3.1%)	1 (.4%)	1 (.4%)	3 (2.2%)	38 (10.2%)	4 (1.4%)	6 (2.8%)	0 (0%)	4 (1.7%)	5 (1.9%)	1 (.3%)	0 (0%)
cervical screening	0 (0%)	0 (0%)	6 (2.5%)	8 (5.8%)	0 (0%)	1 (.4%)	3 (1.4%)	0 (0%)	0 (0%)	1 (.4%)	0 (0%)	1 (.4%)
child welfare (incl. Immunization)	113 (59.2%)	159 (61.9%)	210 (87.5%)	80 (58%)	311 (83.6%)	196 (69.3%)	111 (50.9%)	133 (53%)	173 (73.6%)	194 (73.2%)	184 (61.1%)	158 (62%)
Other	0 (0%)	0 (0%)	1 (.4%)	0 (0%)	0 (0%)	6 (2.1%)	0 (0%)	0 (0%)	0 (0%)	1 (.4%)	0 (0%)	0 (0%)
Any MCH service	72 (37.7%)	98 (38.1%)	38 (15.8%)	48 (34.8%)	101 (27.2%)	66 (23.3%)	127 (58.3%)	121 (48.2%)	47 (20%)	73 (27.5%)	121 (40.2%)	102 (40%)
**Integrated HIV/STI & MCH services received**												
neither	118 (61.8%)	158 (61.5%)	168 (70%)	90 (65.2%)	246 (66.1%)	216 (76.3%)	91 (41.7%)	130 (51.8%)	187 (79.6%)	187 (70.6%)	180 (59.8%)	148 (58%)
MCH only	46 (24.1%)	81 (31.5%)	35 (14.6%)	34 (24.6%)	86 (23.1%)	66 (23.3%)	88 (40.4%)	118 (47%)	39 (16.6%)	51 (19.2%)	121 (40.2%)	100 (39.2%)
HIV/STI only	1 (.5%)	1 (.4%)	34 (14.2%)	0 (0%)	25 (6.7%)	1 (.4%)	0 (0%)	0 (0%)	1 (.4%)	5 (1.9%)	0 (0%)	5 (2%)
HIV/STI & MCH	26 (13.6%)	17 (6.6%)	3 (1.3%)	14 (10.1%)	15 (4%)	0 (0%)	39 (17.9%)	3 (1.2%)	8 (3.4%)	22 (8.3%)	0 (0%)	2 (.8%)
**Receipt of any HIV/STI service plus...**												
Family Planning Provision	20 (10.5%)	13 (5.1%)	2 (.8%)	2 (1.4%)	1 (.3%)	0 (0%)	30 (13.8%)	2 (.8%)	5 (2.1%)	20 (7.5%)	0 (0%)	0 (0%)
Family Planning Counselling	20 (10.5%)	13 (5.1%)	2 (.8%)	1 (.7%)	2 (.5%)	0 (0%)	27 (12.4%)	2 (.8%)	4 (1.7%)	19 (7.2%)	0 (0%)	0 (0%)
ANC	6 (3.1%)	4 (1.6%)	1 (.4%)	4 (2.9%)	12 (3.2%)	0 (0%)	34 (15.6%)	3 (1.2%)	3 (1.3%)	2 (.8%)	0 (0%)	2 (.8%)
PNC (mother or baby)	0 (0%)	0 (0%)	0 (0%)	3 (2.2%)	2 (.5%)	0 (0%)	1 (.5%)	0 (0%)	0 (0%)	0 (0%)	0 (0%)	0 (0%)
cervical screening	0 (0%)	0 (0%)	0 (0%)	6 (4.3%)	0 (0%)	0 (0%)	1 (.5%)	0 (0%)	0 (0%)	1 (.4%)	0 (0%)	0 (0%)
child welfare (incl. Immunization)	1 (.5%)	0 (0%)	35 (14.6%)	0 (0%)	25 (6.7%)	0 (0%)	6 (2.8%)	1 (.4%)	1 (.4%)	12 (4.5%)	0 (0%)	3 (1.2%)
Other RH/MCH service	0 (0%)	0 (0%)	0 (0%)	0 (0%)	0 (0%)	0 (0%)	0 (0%)	0 (0%)	0 (0%)	0 (0%)	0 (0%)	0 (0%)

	District E2 Facilities: Facility i						Facility j					
	Jun2009	Jan2010	Jun2010	Jan2011	Jun2011	Jan2012	Jun2009	Jan2010	Jun2010	Jan2011	Jun2011	Jan2012
**Visits tracked**	139	68	178	157	120	134	126	154	134	197	212	156
**Client age (years)**												
Mean [sd]	26.7 [6.6]	26.9 [6]	26.9 [6.7]	27.5 [7.2]	27.2 [6]	27.4 [7]	25.7 [6.4]	25.8 [6.4]	26.3 [6]	26.7 [6.3]	27 [5.7]	27 [6.8]
Missing	0 (0%)	0 (0%)	0 (0%)	0 (0%)	2 (1.7%)	0 (0%)	0 (0%)	4 (2.6%)	0 (0%)	0 (0%)	2 (.9%)	2 (1.3%)
**Services received per client**												
none	1 (.7%)	2 (2.9%)	0 (0%)	1 (.6%)	1 (.8%)	0 (0%)	13 (10.3%)	4 (2.6%)	1 (.7%)	0 (0%)	4 (1.9%)	0 (0%)
one	61 (43.9%)	25 (36.8%)	78 (43.8%)	70 (44.6%)	42 (35%)	58 (43.3%)	53 (42.1%)	139 (90.3%)	84 (62.7%)	74 (37.6%)	58 (27.4%)	66 (42.3%)
two or more	77 (55.4%)	41 (60.3%)	100 (56.2%)	86 (54.8%)	77 (64.2%)	76 (56.7%)	60 (47.6%)	11 (7.1%)	49 (36.6%)	123 (62.4%)	150 (70.8%)	90 (57.7%)
**Visits where at least one services was received...**												
Mean [sd] services received	1.9 [1]	1.8 [.7]	1.8 [.9]	1.7 [.8]	1.8 [.8]	1.9 [1.2]	1.6 [.6]	1.1 [.4]	1.6 [1]	1.8 [.9]	2 [.9]	2.1 [1.2]
Mean [sd] providers seen	1.3 [.5]	1 [0]	1.2 [.5]	1.2 [.5]	1.2 [.4]	1.2 [.6]	1 [0]	1 [0]	1.3 [.7]	1.6 [.5]	1.7 [.5]	1.5 [.6]
**Top 3 services provided**												
1	Child welfare	Child welfare	Child welfare	Child welfare	Child welfare	Child welfare	Child welfare	Child welfare	Child welfare	Child welfare	Child welfare	Child welfare
2	ANC	ANC	ANC	ANC	ANC	ANC	ANC	ANC	ANC	ANC	ANC	ANC
3	FP provision	HIV counselling	FP provision	FP provision	FP provision	HIV counselling	FP provision	PNC	lab	PNC	FP provision	Other services
**HIV/STI services received**												
HIV testing	17 (12.2%)	5 (7.4%)	6 (3.4%)	1 (.6%)	9 (7.5%)	14 (10.4%)	0 (0%)	0 (0%)	0 (0%)	6 (3%)	6 (2.8%)	2 (1.3%)
HIV counselling	16 (11.5%)	6 (8.8%)	9 (5.1%)	1 (.6%)	10 (8.3%)	16 (11.9%)	1 (.8%)	0 (0%)	0 (0%)	1 (.5%)	3 (1.4%)	0 (0%)
HIV treatment	2 (1.4%)	0 (0%)	0 (0%)	0 (0%)	1 (.8%)	2 (1.5%)	0 (0%)	0 (0%)	2 (1.5%)	1 (.5%)	1 (.5%)	1 (.6%)
HIV PMTCT	0 (0%)	2 (2.9%)	9 (5.1%)	1 (.6%)	3 (2.5%)	1 (.7%)	0 (0%)	0 (0%)	8 (6%)	2 (1%)	3 (1.4%)	3 (1.9%)
Other HIV/STI service	0 (0%)	0 (0%)	0 (0%)	0 (0%)	0 (0%)	0 (0%)	0 (0%)	0 (0%)	0 (0%)	0 (0%)	0 (0%)	0 (0%)
STI counselling or testing	0 (0%)	0 (0%)	1 (.6%)	0 (0%)	0 (0%)	0 (0%)	0 (0%)	0 (0%)	0 (0%)	0 (0%)	0 (0%)	0 (0%)
Any HIV/STI services	22 (15.8%)	6 (8.8%)	10 (5.6%)	1 (.6%)	12 (10%)	17 (12.7%)	1 (.8%)	0 (0%)	2 (1.5%)	8 (4.1%)	7 (3.3%)	2 (1.3%)
**MCH services received**												
Family Planning Provision	27 (19.4%)	4 (5.9%)	23 (12.9%)	22 (14%)	14 (11.7%)	13 (9.7%)	21 (16.7%)	6 (3.9%)	16 (11.9%)	12 (6.1%)	15 (7.1%)	14 (9%)
Family Planning Counselling	15 (10.8%)	2 (2.9%)	17 (9.6%)	7 (4.5%)	12 (10%)	8 (6%)	6 (4.8%)	2 (1.3%)	7 (5.2%)	10 (5.1%)	10 (4.7%)	7 (4.5%)
ANC	39 (28.1%)	15 (22.1%)	41 (23%)	32 (20.4%)	33 (27.5%)	56 (41.8%)	30 (23.8%)	37 (24%)	42 (31.3%)	28 (14.2%)	37 (17.5%)	30 (19.2%)
PNC (mother or baby)	4 (2.9%)	1 (1.5%)	2 (1.1%)	1 (.6%)	0 (0%)	0 (0%)	2 (1.6%)	4 (2.6%)	4 (3%)	15 (7.6%)	6 (2.8%)	2 (1.3%)
cervical screening	0 (0%)	0 (0%)	1 (.6%)	1 (.6%)	0 (0%)	1 (.7%)	0 (0%)	0 (0%)	0 (0%)	0 (0%)	0 (0%)	0 (0%)
child welfare (incl. Immunization)	72 (51.8%)	47 (69.1%)	112 (62.9%)	104 (66.2%)	66 (55%)	80 (59.7%)	61 (48.4%)	96 (62.3%)	76 (56.7%)	149 (75.6%)	160 (75.5%)	119 (76.3%)
Other	2 (1.4%)	1 (1.5%)	0 (0%)	0 (0%)	0 (0%)	1 (.7%)	0 (0%)	0 (0%)	0 (0%)	0 (0%)	0 (0%)	0 (0%)
Any MCH service	72 (51.8%)	20 (29.4%)	71 (39.9%)	56 (35.7%)	51 (42.5%)	74 (55.2%)	54 (42.9%)	48 (31.2%)	63 (47%)	60 (30.5%)	61 (28.8%)	48 (30.8%)
**Integrated HIV/STI & MCH services received**												
neither	64 (46%)	48 (70.6%)	105 (59%)	101 (64.3%)	66 (55%)	59 (44%)	72 (57.1%)	106 (68.8%)	70 (52.2%)	137 (69.5%)	150 (70.8%)	107 (68.6%)
MCH only	53 (38.1%)	14 (20.6%)	63 (35.4%)	55 (35%)	42 (35%)	58 (43.3%)	53 (42.1%)	48 (31.2%)	62 (46.3%)	52 (26.4%)	55 (25.9%)	47 (30.1%)
HIV/STI only	3 (2.2%)	0 (0%)	2 (1.1%)	0 (0%)	3 (2.5%)	1 (.7%)	0 (0%)	0 (0%)	1 (.7%)	0 (0%)	1 (.5%)	1 (.6%)
HIV/STI & MCH	19 (13.7%)	6 (8.8%)	8 (4.5%)	1 (.6%)	9 (7.5%)	16 (11.9%)	1 (.8%)	0 (0%)	1 (.7%)	8 (4.1%)	6 (2.8%)	1 (.6%)
**Receipt of any HIV/STI service plus...**												
Family Planning Provision	4 (2.9%)	0 (0%)	2 (1.1%)	1 (.6%)	1 (.8%)	1 (.7%)	0 (0%)	0 (0%)	0 (0%)	0 (0%)	3 (1.4%)	0 (0%)
Family Planning Counselling	2 (1.4%)	0 (0%)	1 (.6%)	0 (0%)	0 (0%)	1 (.7%)	0 (0%)	0 (0%)	0 (0%)	1 (.5%)	0 (0%)	0 (0%)
ANC	15 (10.8%)	6 (8.8%)	6 (3.4%)	0 (0%)	8 (6.7%)	15 (11.2%)	1 (.8%)	0 (0%)	1 (.7%)	6 (3%)	3 (1.4%)	1 (.6%)
PNC (mother or baby)	0 (0%)	0 (0%)	0 (0%)	0 (0%)	0 (0%)	0 (0%)	0 (0%)	0 (0%)	0 (0%)	1 (.5%)	0 (0%)	0 (0%)
cervical screening	0 (0%)	0 (0%)	0 (0%)	0 (0%)	0 (0%)	0 (0%)	0 (0%)	0 (0%)	0 (0%)	0 (0%)	0 (0%)	0 (0%)
child welfare (incl. Immunization)	3 (2.2%)	0 (0%)	2 (1.1%)	0 (0%)	2 (1.7%)	0 (0%)	0 (0%)	0 (0%)	1 (.7%)	1 (.5%)	0 (0%)	0 (0%)
Other RH/MCH service	1 (.7%)	0 (0%)	0 (0%)	0 (0%)	0 (0%)	1 (.7%)	0 (0%)	0 (0%)	0 (0%)	0 (0%)	0 (0%)	0 (0%)

	District E2 Facilities: Facility k						Facility l					
	Jun2009	Jan2010	Jun2010	Jan2011	Jun2011	Jan2012	Jun2009	Jan2010	Jun2010	Jan2011	Jun2011	Jan2012
**Visits tracked**	230	131	143	135	135	217	169	153	162	171	209	107
**Client age (years)**												
Mean [sd]	28.7 [10.6]	26.6 [5.8]	26.7 [5.6]	27.5 [6.8]	28.2 [6.7]	28.6 [8.5]	27.3 [6.5]	29 [7.3]	29.1 [7.3]	27.3 [6.3]	28.5 [6.4]	27.6 [6.2]
Missing	0 (0%)	0 (0%)	0 (0%)	1 (.7%)	2 (1.5%)	2 (.9%)	0 (0%)	4 (2.6%)	0 (0%)	3 (1.8%)	1 (.5%)	4 (3.7%)
**Services received per client**												
none	2 (.9%)	0 (0%)	0 (0%)	0 (0%)	1 (.7%)	0 (0%)	0 (0%)	1 (.7%)	0 (0%)	0 (0%)	0 (0%)	0 (0%)
one	127 (55.2%)	62 (47.3%)	30 (21%)	106 (78.5%)	77 (57%)	134 (61.8%)	73 (43.2%)	83 (54.2%)	47 (29%)	78 (45.6%)	75 (35.9%)	21 (19.6%)
two or more	101 (43.9%)	69 (52.7%)	113 (79%)	29 (21.5%)	57 (42.2%)	83 (38.2%)	96 (56.8%)	69 (45.1%)	115 (71%)	93 (54.4%)	134 (64.1%)	86 (80.4%)
**Visits where at least one services was received...**												
Mean [sd] services received	1.7 [1]	2 [1.4]	3.3 [1.8]	1.3 [.8]	1.4 [.5]	1.6 [.9]	1.8 [1]	1.8 [1]	2.6 [1.6]	1.9 [1]	1.8 [.7]	2.6 [1.2]
Mean [sd] providers seen	1 [.2]	1.4 [.6]	1.7 [.5]	1.1 [.3]	1.1 [.3]	1.5 [.7]	1.1 [.3]	1.2 [.4]	1.4 [.6]	1.3 [.5]	1.7 [.6]	1.9 [.7]
**Top 3 services provided**												
1	Child welfare	Child welfare	Child welfare	Child welfare	Child welfare	Child welfare	Child welfare	Child welfare	Child welfare	Other services	Child welfare	Child welfare
2	FP provision	ANC	ANC	ANC	ANC	ANC	ANC	ANC	ANC	Child welfare	Other services	ANC
3	ANC	FP provision	general counselling	FP provision	FP provision	Other services	FP provision	Other services	general counselling	ANC	ANC	Other services
**HIV/STI services received**												
HIV testing	29 (12.6%)	12 (9.2%)	23 (16.1%)	2 (1.5%)	0 (0%)	2 (.9%)	15 (8.9%)	14 (9.2%)	11 (6.8%)	3 (1.8%)	0 (0%)	10 (9.3%)
HIV counselling	31 (13.5%)	14 (10.7%)	17 (11.9%)	1 (.7%)	1 (.7%)	2 (.9%)	15 (8.9%)	13 (8.5%)	13 (8%)	1 (.6%)	2 (1%)	7 (6.5%)
HIV treatment	1 (.4%)	1 (.8%)	0 (0%)	0 (0%)	0 (0%)	0 (0%)	0 (0%)	1 (.7%)	2 (1.2%)	2 (1.2%)	10 (4.8%)	0 (0%)
HIV PMTCT	0 (0%)	0 (0%)	17 (11.9%)	3 (2.2%)	2 (1.5%)	2 (.9%)	0 (0%)	18 (11.8%)	5 (3.1%)	0 (0%)	0 (0%)	0 (0%)
Other HIV/STI service	0 (0%)	0 (0%)	0 (0%)	0 (0%)	0 (0%)	0 (0%)	0 (0%)	0 (0%)	1 (.6%)	0 (0%)	0 (0%)	0 (0%)
STI counselling or testing	2 (.9%)	0 (0%)	0 (0%)	1 (.7%)	0 (0%)	0 (0%)	2 (1.2%)	1 (.7%)	3 (1.9%)	1 (.6%)	0 (0%)	0 (0%)
Any HIV/STI services	34 (14.8%)	14 (10.7%)	23 (16.1%)	3 (2.2%)	1 (.7%)	2 (.9%)	17 (10.1%)	18 (11.8%)	15 (9.3%)	5 (2.9%)	10 (4.8%)	10 (9.3%)
**MCH services received**												
Family Planning Provision	54 (23.5%)	27 (20.6%)	23 (16.1%)	25 (18.5%)	17 (12.6%)	21 (9.7%)	19 (11.2%)	38 (24.8%)	23 (14.2%)	34 (19.9%)	21 (10%)	18 (16.8%)
Family Planning Counselling	39 (17%)	23 (17.6%)	14 (9.8%)	8 (5.9%)	3 (2.2%)	4 (1.8%)	16 (9.5%)	6 (3.9%)	31 (19.1%)	16 (9.4%)	15 (7.2%)	11 (10.3%)
ANC	51 (22.2%)	29 (22.1%)	33 (23.1%)	27 (20%)	25 (18.5%)	39 (18%)	35 (20.7%)	40 (26.1%)	46 (28.4%)	46 (26.9%)	39 (18.7%)	22 (20.6%)
PNC (mother or baby)	6 (2.6%)	1 (.8%)	23 (16.1%)	5 (3.7%)	1 (.7%)	4 (1.8%)	2 (1.2%)	0 (0%)	6 (3.7%)	12 (7%)	1 (.5%)	2 (1.9%)
cervical screening	0 (0%)	0 (0%)	0 (0%)	0 (0%)	0 (0%)	0 (0%)	0 (0%)	1 (.7%)	0 (0%)	0 (0%)	1 (.5%)	0 (0%)
child welfare (incl. Immunization)	92 (40%)	77 (58.8%)	90 (62.9%)	81 (60%)	83 (61.5%)	147 (67.7%)	122 (72.2%)	68 (44.4%)	110 (67.9%)	51 (29.8%)	127 (60.8%)	71 (66.4%)
Other	6 (2.6%)	0 (0%)	0 (0%)	0 (0%)	0 (0%)	0 (0%)	0 (0%)	1 (.7%)	0 (0%)	0 (0%)	0 (0%)	0 (0%)
Any MCH service	116 (50.4%)	58 (44.3%)	77 (53.8%)	57 (42.2%)	44 (32.6%)	66 (30.4%)	55 (32.5%)	80 (52.3%)	82 (50.6%)	96 (56.1%)	64 (30.6%)	43 (40.2%)
**Integrated HIV/STI & MCH services received**												
neither	106 (46.1%)	73 (55.7%)	57 (39.9%)	77 (57%)	90 (66.7%)	151 (69.6%)	113 (66.9%)	69 (45.1%)	80 (49.4%)	74 (43.3%)	136 (65.1%)	62 (57.9%)
MCH only	90 (39.1%)	44 (33.6%)	63 (44.1%)	55 (40.7%)	44 (32.6%)	64 (29.5%)	39 (23.1%)	66 (43.1%)	67 (41.4%)	92 (53.8%)	63 (30.1%)	35 (32.7%)
HIV/STI only	8 (3.5%)	0 (0%)	9 (6.3%)	1 (.7%)	1 (.7%)	0 (0%)	1 (.6%)	4 (2.6%)	0 (0%)	1 (.6%)	9 (4.3%)	2 (1.9%)
HIV/STI & MCH	26 (11.3%)	14 (10.7%)	14 (9.8%)	2 (1.5%)	0 (0%)	2 (.9%)	16 (9.5%)	14 (9.2%)	15 (9.3%)	4 (2.3%)	1 (.5%)	8 (7.5%)
**Receipt of any HIV/STI service plus...**												
Family Planning Provision	5 (2.2%)	0 (0%)	3 (2.1%)	0 (0%)	0 (0%)	0 (0%)	12 (7.1%)	13 (8.5%)	8 (4.9%)	3 (1.8%)	0 (0%)	4 (3.7%)
Family Planning Counselling	5 (2.2%)	0 (0%)	2 (1.4%)	0 (0%)	0 (0%)	0 (0%)	9 (5.3%)	2 (1.3%)	9 (5.6%)	0 (0%)	0 (0%)	2 (1.9%)
ANC	21 (9.1%)	14 (10.7%)	10 (7%)	2 (1.5%)	0 (0%)	2 (.9%)	4 (2.4%)	1 (.7%)	6 (3.7%)	1 (.6%)	1 (.5%)	4 (3.7%)
PNC (mother or baby)	0 (0%)	0 (0%)	2 (1.4%)	0 (0%)	0 (0%)	0 (0%)	1 (.6%)	0 (0%)	3 (1.9%)	0 (0%)	0 (0%)	0 (0%)
cervical screening	0 (0%)	0 (0%)	0 (0%)	0 (0%)	0 (0%)	0 (0%)	0 (0%)	0 (0%)	0 (0%)	0 (0%)	0 (0%)	0 (0%)
child welfare (incl. Immunization)	3 (1.3%)	0 (0%)	11 (7.7%)	0 (0%)	0 (0%)	0 (0%)	4 (2.4%)	2 (1.3%)	3 (1.9%)	0 (0%)	0 (0%)	1 (.9%)
Other RH/MCH service	0 (0%)	0 (0%)	0 (0%)	0 (0%)	0 (0%)	0 (0%)	0 (0%)	0 (0%)	0 (0%)	0 (0%)	0 (0%)	0 (0%)
